# A Bacterial Sulfotransferase Catalyzes an Unusual Di‐Sulfation in Natural Products Biosynthesis

**DOI:** 10.1002/cbic.202500024

**Published:** 2025-04-04

**Authors:** Conor Pulliam, Lukuan Hou, Dan Xue, Mingming Xu, Katherine Holandez‐Lopez, Jie Li

**Affiliations:** ^1^ Department of Chemistry and Biochemistry University of South Carolina Columbia SC 29208 USA

**Keywords:** sulfotransferases, adipostatin, di‐sulfation, PAPS, *Streptomyces*

## Abstract

Sulfation is a widely used strategy in nature to modify the solubility, polarity, and biological activities of molecules. The enzymes catalyzing sulfation, sulfotransferases (STs), are typically highly specific to a single sulfation site in a molecule. Herein, the identification and characterization of sulfated adipostatins is reported and reveals a novel sulfotransferase, AdpST, which is responsible for di‐sulfation at two sites of adipostatins. The initial bioinformatic analysis in search of adipostatin analogs from *Streptomyces davaonensis* DSM101723 identifies *adpST* and a 3’‐phosphoadenosine‐5’‐phosphosulfate (PAPS) biosynthetic cassette, which are co‐clustered with the adipostatin‐encoding type III polyketide synthase. Mono‐ and di‐sulfated adipostatin analogs are discovered in the extracts of *S. davaonensis* DSM101723, whereas di‐sulfated bacterial natural products has not been reported. Using a series of in vivo and in vitro experiments, it is confirmed that AdpST is solely responsible for both mono‐ and di‐sulfation of adipostatins, a catalytic activity which has not been identified in bacterial PAPS‐dependent STs to date. It is further demonstrated that the dedicated PAPS biosynthetic cassette improves di‐sulfation capacity. Lastly, it is determined that AdpST shares similarity with a small group of uncharacterized STs, suggesting the presence of additional unique bacterial STs in nature, and that AdpST is phylogenetically distant from many characterized STs.

## Graphical abstract

Herein, a unique sulfotransferase, AdpST, is identified from the *adp* biosynthetic gene clusters (BGC) in *Streptomyces davaonensis* DSM101723 and demonstrates that AdpST possesses di‐sulfation activity, which is unprecedented for bacterial phosphoadenosine‐5’‐phosphosulfate (PAPS)‐dependent sulfotransferases. It is further shown that the *adp* BGC contains a dedicated PAPS biosynthetic cassette essential for improving sulfation capacity.

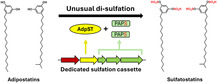

## 1 Introduction

Sulfur is a ubiquitous element in nature that is present in many essential biomolecules, as well as in many natural products. Sulfur moieties provide immense chemical diversity in sulfur‐containing natural products^[^ ^1^, ^2^ ^]^ and can directly contribute to their biological activities, such as antimicrobial and antioxidant activities.^[^ ^3^, ^4^, ^5^, ^6^ ^]^ One of these sulfur‐based chemical groups, sulfate, is common in a variety of macromolecules and primary metabolites, including saccharides such as heparin and heparan sulfate,^[^ ^7^ ^]^ lipids such as cholesterol sulfate,^[^ ^8^ ^]^ and O‐sulfated tyrosyl residues in proteins.^[^ ^9^, ^10^ ^]^ However, sulfation of secondary metabolites is understudied^[^ ^11^ ^]^ and requires further investigation to reveal the chemical diversity that sulfation can provide for natural products.

**Figure 1:**
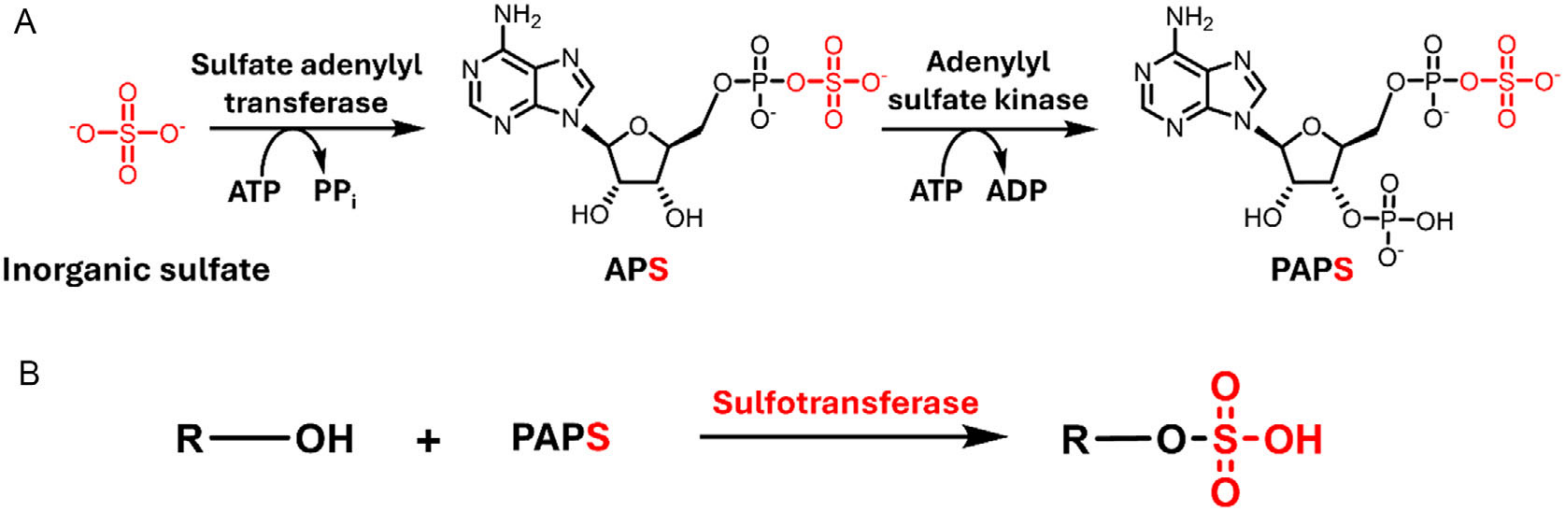
A) Biosynthetic pathway of the PAPS sulfate donor substrate involves two enzymes, a sulfate adenylyl transferase and an adenylyl sulfate kinase. B) Following PAPS biosynthesis, the transfer of a sulfate group from PAPS to an acceptor hydroxyl group is carried out by a sulfotransferase.

Across the domains of life, sulfation is performed by enzymes called sulfotransferases (STs), which catalyze the transfer of an SO_3_ group from a donor substrate to the hydroxyl of an acceptor substrate, resulting in an O‐sulfated product.^[^ ^12^, ^13^ ^]^ The donor substrate for sulfation reactions is most often a derivative of adenosine triphosphate (ATP) called 3’‐phosphoadenosine‐5’‐phosphosulfate (PAPS).^[^ ^14^, ^15^ ^]^ STs that utilize PAPS as the sulfate donor substrate are termed PAPS‐dependent STs; for the purposes of this study, any mention of STs will refer to PAPS‐dependent STs unless otherwise stated. Whereas much sulfation research has focused on the activities of eukaryotic STs in metabolite modification, solubilization, and detoxification,^[^ ^8^, ^13^, ^14^, ^15^ ^]^ a distinct knowledge gap in the understanding of prokaryotic STs remains. In bacteria, biosynthesis of the PAPS sulfate donor substrate first involves a sulfate adenylyltransferase that catalyzes the nucleophilic attack of inorganic sulfate onto the α‐phosphate of ATP, producing adenosine‐5’‐phosphosulfate (APS).^[^ ^16^ ^]^ In bacterial PAPS biosynthetic pathways, this sulfate adenylyltransferase may consist of two proteins, a large and a small subunit.^[^ ^17^ ^]^ Following formation of APS by sulfate adenylyltransferase, an adenylylsulfate kinase phosphorylates the 5’‐hydroxyl of APS in an ATP‐dependent manner to generate PAPS.^[^ ^15^, ^16^ ^]^ An overview of the PAPS biosynthetic pathway is shown in **Figure** **1**. A given genome may contain only one or a few copies of PAPS biosynthetic genes. In the human genome, for example, only two genes encoding PAPS biosynthetic enzymes are known, *PAPSS1* and *PAPSS2*, which serve to generate PAPS for all PAPS‐dependent metabolic pathways.^[^ ^15^ ^]^ Even for bacteria, genes encoding PAPS biosynthetic enzymes are often present in few copies and similarly serve as a communal source of PAPS throughout the cell. Thus, whereas bacteria often use biosynthetic gene clusters (BGCs) to coordinate the expression of genes sharing a common biosynthetic pathway,^[^ ^18^ ^]^ a BGC encoding a sulfation pathway is likely to encode a pathway‐specific ST but generally will not encode a dedicated PAPS biosynthetic cassette.^[^ ^11^, ^19^, ^20^ ^]^ Such pathways will utilize PAPS generated from enzymes encoded elsewhere in the genome.

Herein, we expand on our previous discovery^[^ ^21^ ^]^ of adipostatin analogs—a class of alkylresorcinol natural products^[^ ^22^ ^]^—from *S. davaonensis* DSM101723 by identifying and characterizing adipostatin analogs with mono‐ or di‐sulfated resorcinol head groups. Whereas mono‐sulfated bacterial natural products have been reported frequently, di‐sulfated bacterial natural products have not been identified previously to our knowledge.^[^ ^11^, ^17^, ^20^, ^23^, ^24^ ^]^ We further demonstrate that both the mono‐ and di‐sulfated analogs are sulfated by a single ST, AdpST. Although di‐sulfation has been observed for bacterial PAPS‐independent arylsulfate sulfotransferases (ASSTs) in the in vitro enzymatic synthesis of sulfated plant polyphenols,^[^ ^25^, ^26^ ^]^ this activity has not been observed for bacterial PAPS‐dependent STs to our knowledge, including both STs from primary metabolism and STs from secondary metabolism.^[^ ^11^, ^17^, ^20^, ^23^, ^24^, ^27^, ^28^, ^29^, ^30^ ^]^ Furthermore, in contrast to the majority of bacterial ST‐containing BGCs, we confirm that the BGC encoding the sulfated adipostatin analogs contains a dedicated PAPS biosynthetic cassette and demonstrate that this dedicated cassette is important for ensuring sufficient sulfate donor substrate is present to support mono‐ and di‐sulfation of adipostatins. Lastly, we perform a phylogenetic characterization and sequence similarity analysis to demonstrate the sequence divergence of AdpST compared to known bacterial STs and to show that other bacterial STs with di‐sulfation activities likely exist in nature.

## 2 Results and Discussion

### 2.1 Bioinformatic Analysis Identifies a “Sulfation Cassette” in the *adp* BGC

**Figure 2:**
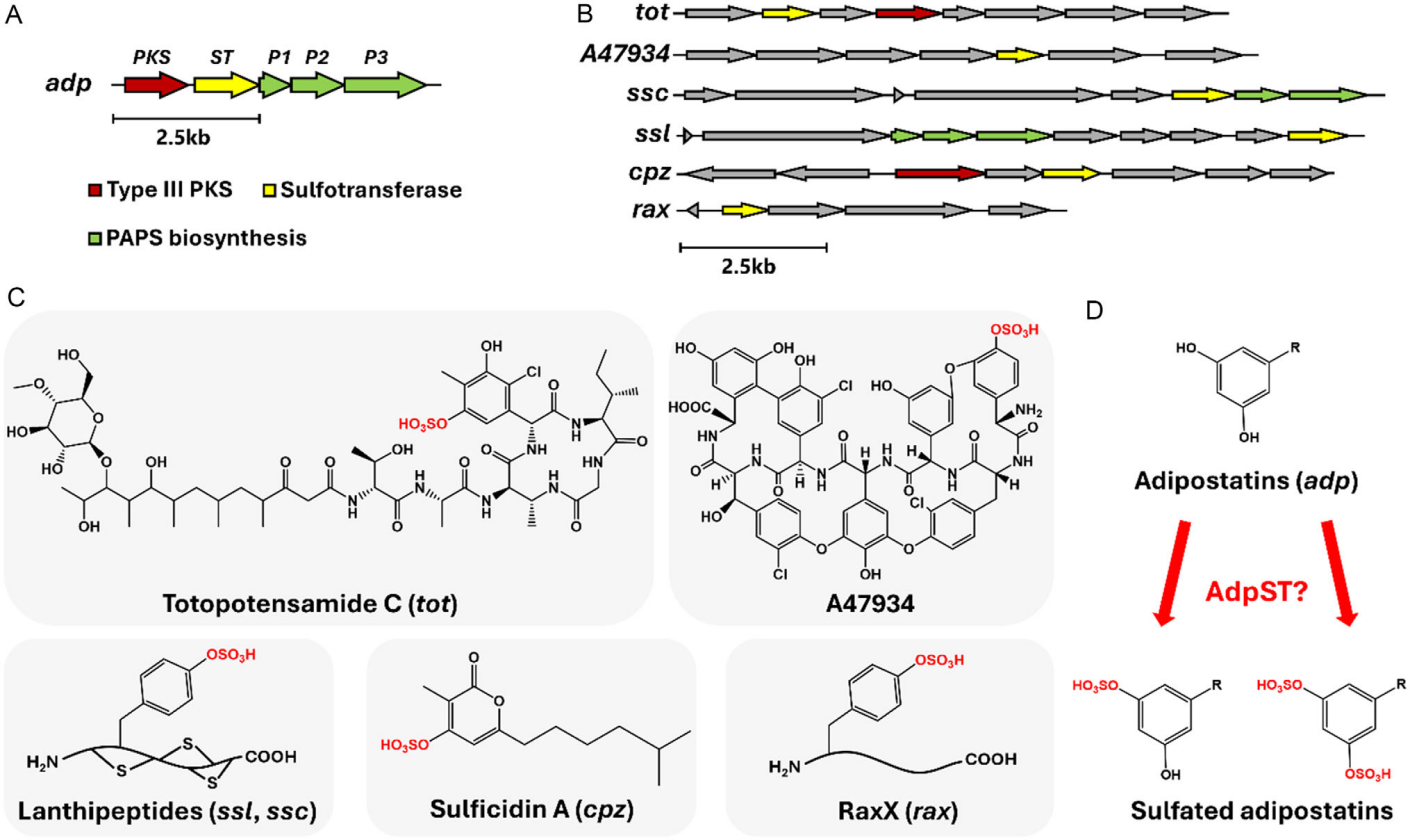
A) Identification of a “sulfation cassette” in the *adp* BGC, consisting of a type III polyketide synthase (*adpPKS*), a single sulfotransferase (*adpST*), and a PAPS biosynthetic cassette (*adpP1*, *adpP2*, *adpP3*). B) Previously reported bacterial sulfotransferase‐containing BGCs. Type III polyketide synthases are colored in red, sulfotransferases in yellow, and PAPS biosynthetic genes in green. C) Chemical structures of the gene products of previously reported sulfotransferase‐containing BGCs. Note the presence of only mono‐sulfation in previously reported metabolites. D) Hypothesized formation of sulfated adipostatin analogs by AdpST encoded in the *adp* BGC.

Based on our previous chemical analysis of the extracts of *S. davaonensis* DSM101723,^[^ ^21^ ^]^ we envisioned that this organism may produce additional adipostatin analogs. To explore this hypothesis, we performed a bioinformatic analysis of the *S. davaonensis* DSM101723 genome (NCBI accession ASM34932v1) focused around the type III polyketide synthase (T3PKS) gene, *adpPKS*, responsible for production of adipostatins that we identified from our previous study. Intriguingly, we found that *adpPKS* is present as part of a five‐gene operon which we termed the *adp* BGC, containing *adpPKS* along with a putative ST‐encoding gene which we named *adpST*. The remaining three genes in the *adp* BGC downstream of *adpST* were predicted to encode an adenylylsulfate kinase (*adpP1*), a sulfate adenylyltransferase small subunit (*adpP2*), and a sulfate adenylyltransferase large subunit (*adpP3*), respectively (**Figure** **2**A). We recognized *adpP1*, *adpP2,* and *adpP3* to encode the biosynthetic enzymes needed to produce PAPS, the most common sulfate donor substrate for STs. This was additionally interesting, as this suggested that *adpP1*, *adpP2*, and *adpP3* constituted a dedicated PAPS biosynthetic cassette that is co‐expressed with *adpPKS* and *adpST*, creating a “sulfation cassette” with all genes necessary to make the sulfated adipostatin gene products. This is distinct from the commonly observed use of a communal PAPS biosynthetic cassette in bacteria that serves the needs of multiple biosynthetic pathways. To confirm that *adpP1*, *adpP2*, and *adpP3* indeed comprised a dedicated PAPS biosynthetic cassette, we performed a protein BLAST search of *adpP1*, *adpP2*, and *adpP3* against the *S. davaonensis* DSM101723 genome to identify other PAPS biosynthetic enzymes, from which we found two other sets of PAPS biosynthetic enzymes in the genome (Extended Data File S1, Supporting Information). This suggested that these other PAPS biosynthetic cassettes likely serve to produce communal PAPS for use in multiple biosynthetic pathways, whereas the PAPS biosynthetic cassette in the *adp* BGC is co‐expressed with *adpPKS* and *adpST* to ensure there is a sufficient quantity of PAPS in the cell to support adipostatin sulfation.

Intrigued that the *adp* BGC encoded a ST co‐clustered with a PAPS biosynthetic cassette, we performed an exhaustive literature search to compare the *adp* BGC to other bacterial secondary metabolic ST‐encoding BGCs. From our search,^[^ ^11^, ^17^, ^19^, ^20^, ^24^ ^]^ we identified six ST‐encoding BGCs (Figure 2B), two of which (*cpz*, *tot*) encoded a T3PKS and another two recently reported BGCs (*ssl*, *ssc*) that encoded a partial or complete dedicated PAPS biosynthetic cassette. However, none of these BGCs encoded both a T3PKS and a dedicated PAPS biosynthetic cassette. This suggested that the *adp* BGC, containing both a T3PKS and a dedicated PAPS biosynthetic cassette, may indeed encode additional adipostatin analogs with unique chemical structures. We further explored the chemical structures of the gene products from previously reported ST‐containing BGCs and noted that each sulfated gene product of these BGCs was sulfated on a single phenolic hydroxyl group, despite the presence of multiple phenolic hydroxyl groups with the potential to serve as a sulfation site in some of the compounds (Figure 2C). Based on these structures, we hypothesized that the ST encoded in the *adp* BGC, AdpST, may sulfate the phenolic hydroxyl group(s) of adipostatins to generate sulfated analogs (Figure 2D).

### 2.2 Isolation and Structural Characterization of Unknown Mass Features Leads to the Identification of Sulfatostatins

**Figure 3:**
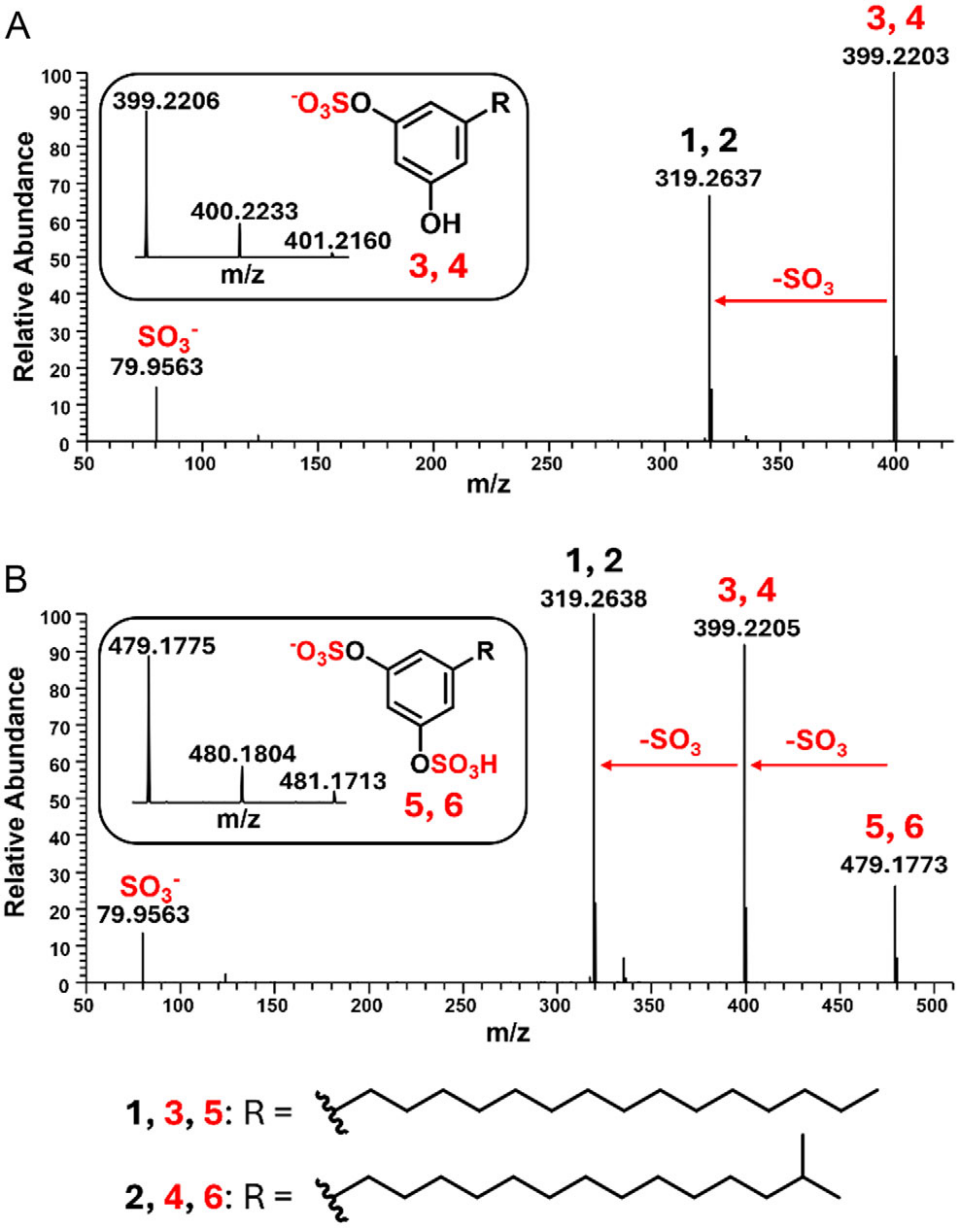
MS and MS/MS characterization of isolated sulfatostatins suggests the presence of both mono‐ and di‐sulfated analogs. A) In the MS/MS spectrum of **3**/**4**, a loss of one SO_3_ to **1**/**2** is observed. B) In the MS/MS spectrum of **5**/**6**, a loss of one SO_3_ to **3**/**4** or two SO_3_ to **1**/**2** are both observed. The corresponding MS spectrum of each MS/MS spectrum is shown in a rounded box.

We began our investigation of adipostatin analogs using liquid chromatography‐high resolution mass spectrometry (MS) to analyze crude extracts of *S. davaonensis* DSM101723. We first targeted the most abundant adipostatin A (**1**) and adipostatin B (**2**) as a baseline for our investigation of sulfated adipostatin analogs (Figure S1, Supporting Information), which exhibit [M‐H]^−^ *m*/*z* values of 319.2643 and 319.2644, representing errors of 1.9 and 2.2 ppm, respectively (theoretical *m*/*z* = 319.2637). We then targeted *m*/*z* values of 399.22, corresponding to a 79.96 Da mass increase compared to **1** or **2**, which would result from the addition of an SO_3_ group onto one of the phenolic hydroxyl groups of **1** or **2**. Using this strategy, we identified two additional sets of chromatographic peaks exhibiting similar elution profiles to **1** and **2** with *m*/*z* values of 399.2208 and 399.2205 (Figure S2, Supporting Information), representing errors of 0.7 and −0.1 ppm, respectively (theoretical *m*/*z* = 399.2205). We also searched for an *m*/*z* value corresponding to di‐sulfated analogs of **1** and **2** due to the symmetry of the hydroxyl groups of **1** and **2**, which we hypothesized may give each hydroxyl group an equal probability of being sulfated. We indeed identified another set of chromatographic peaks with [M‐H]^−^ *m*/*z* values of 479.1778 and 479.1774 (Figure S3, Supporting Information), representing errors of 1.0 and 0.1 ppm, respectively (theoretical *m*/*z* = 479.1773). Thus, based on the similar elution profiles of these peaks to **1** and **2** and the mass increases compared to **1** and **2**, we hypothesized that the uncharacterized mass features corresponded to mono‐ and di‐sulfated analogs of **1** and **2**, with sulfation occurring on one or both of the resorcinol hydroxyls. This was particularly interesting, as di‐sulfated and tri‐sulfated plant metabolites have been reported before,^[^ ^25^, ^26^ ^]^ but di‐sulfated bacterial secondary metabolites have not been observed previously to our knowledge. We named the mono‐sulfated analogs sulfatostatin A (**3**) and sulfatostatin B (**4**) and the di‐sulfated analogs sulfatostatin C (**5**) and sulfatostatin D (**6**). We next attempted to isolate and structurally characterize the putative sulfated adipostatin analogs via preparative high‐performance liquid chromatography (HPLC), utilizing high‐resolution MS to guide the isolation. We were able to isolate enough **1**, **2**, and **6** for nuclear magnetic resonance (NMR) spectroscopy structural characterization (Figure S4–S9 and Table S1–S3, Supporting Information). We also analyzed the presence of mono‐ or di‐sulfation of **3**–**6** via high‐resolution tandem mass spectrometry (MS/MS). MS/MS analysis of **3**–**6** produced diagnostic fragmentation patterns resulting from a neutral loss of one or two SO_3_ groups. In the MS/MS spectrum of **3** and **4**, we observed fragment ions with *m*/*z* values matching those of **1** and **2**, in addition to a fragment at *m*/*z* = 79.9563 (theoretical *m*/*z* = 79.9568, −6.5 ppm), corresponding to SO_3_^−^ (**Figure** **3**A). In the MS/MS spectrum of **5** and **6**, we observed fragment ions with *m*/*z* values matching **3** and **4** and fragments with *m*/*z* values matching **1** and **2**, in addition to a fragment at *m*/*z* = 79.9563, which similarly corresponded to the SO_3_^−^ ion that was also observed in the MS/MS spectrum of **3** and **4** (Figure 3B). Taking into account our NMR and MS/MS analyses, we were able to confirm that the isolated compounds **3**–**6** were indeed sulfated analogs of **1** and **2**.

### 2.3 AdpST is Solely Responsible for Mono‐ and Di‐Sulfation of Adipostatins

**Figure 4:**
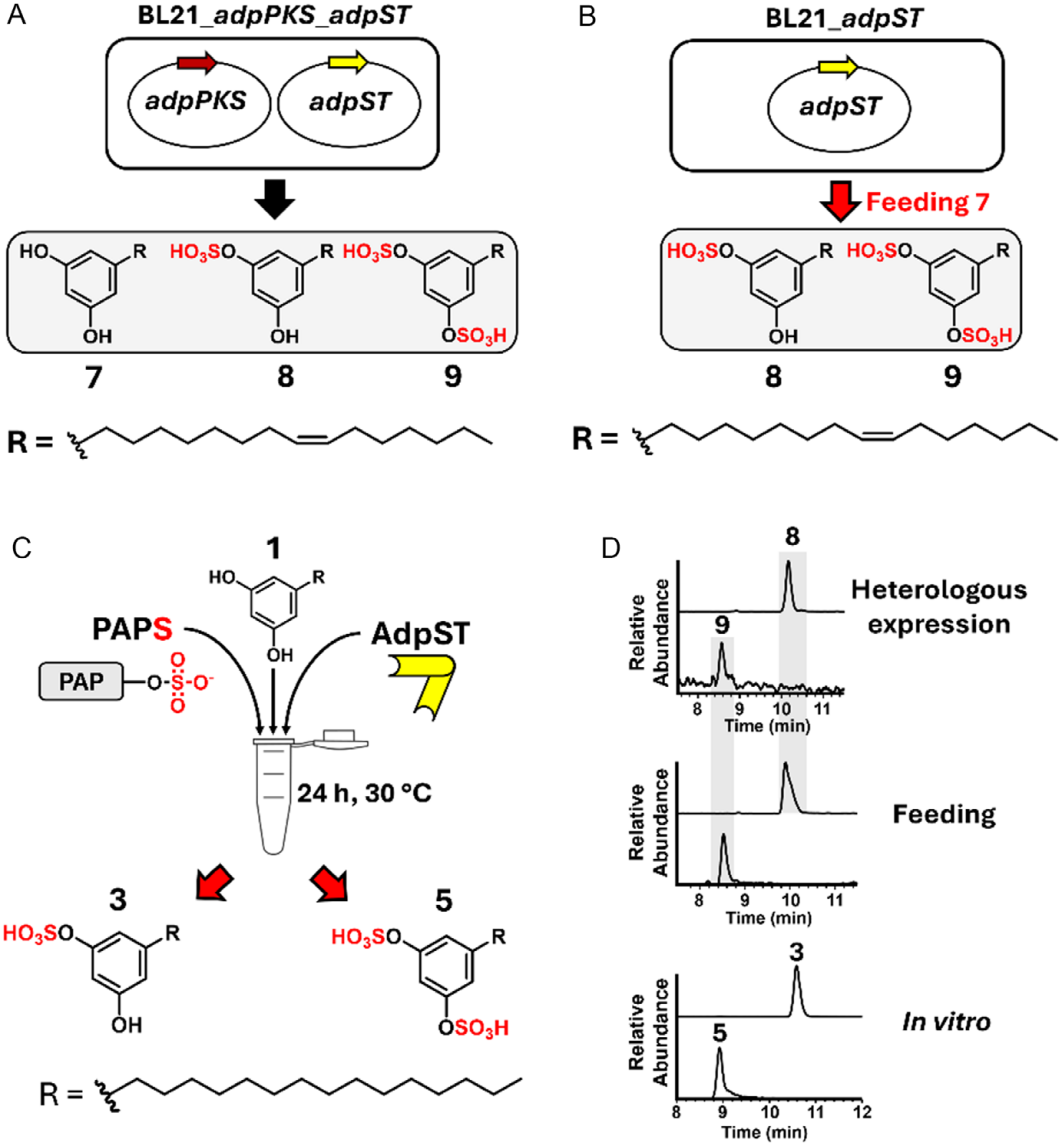
A) Heterologous expression of sulfated adipostatins in *E. coli* BL21 connects the *adp* BGC to sulfatostatin production. B) Feeding of **7** to *E. coli* BL21 expressing *adpST* resulted in the production of **8** and **9**. C) Purified AdpST is able to convert **1** into the mono‐sulfated **3** and di‐sulfated **5** in vitro. D) Extracted ion chromatograms (EICs) of the results from the experiments depicted in (A–C).

Given that AdpST appeared to be the sole ST encoded in the *adp* BGC and that di‐sulfated adipostatin analogs were present in *S. davaonensis* DSM101723 crude extracts, we next sought to determine if AdpST is solely responsible for adipostatin mono‐ and di‐sulfation. We started by heterologously expressing *adpPKS* and *adpST* in *E. coli* BL21 (BL21_*adpPKS*_*adpST*). MS analysis of crude extracts of BL21_*adpPKS*_*adpST* revealed the production of new compounds not identified in extracts of the native *S. davaonensis* DSM101723, with *m*/*z* values of 317.25 (**7**), 397.25 (**8**), and 477.25 (**9**) (Figure S10, Supporting Information). These compounds were 2 Da in mass lower than the corresponding native adipostatin analogs from *S. davaonensis* DSM101723, likely due to a single unsaturation in the aliphatic chain. We expected that **7** was a known adipostatin analog named bilobol because, in our previous study, the only compound identified from the expression of *adpPKS* in *E. coli* BL21 with an *m*/*z* value of 317.25 was bilobol.^[^ ^21^ ^]^ Thus, we expected that **8** and **9** were mono‐ and di‐sulfated analogs of bilobol (**7**), respectively. To confirm that the observed ion at *m*/*z* 317.25 was indeed bilobol, we scaled up the cultivation and isolated **7**. NMR spectroscopic analysis of **7** and comparison to the NMR spectroscopic analysis of bilobol from our previous study^[^ ^21^ ^]^ then confirmed the identity of **7** as bilobol (Figure S11, S12, and Table S4, Supporting Information). We subsequently performed an MS/MS analysis of **8** and **9**, resulting in a loss of 80 Da from **8** and a loss of 160 Da from **9** and both producing a fragment ion corresponding to **7**. This observation, combined with our NMR characterization of **7**, indicated that **8** and **9** were mono‐ and di‐sulfated products of **7** (**Figure** **4**A). Our BL21_*adpPKS*_*adpST* expression system provided the first experimental evidence connecting *adpPKS*/*adpST* expression to sulfated adipostatin production. To further support the role of AdpST as the sole ST responsible for di‐sulfation, we constructed a new *E. coli* BL21 expression strain expressing only *adpST* (BL21_*adpST*), followed by feeding this strain with isolated compound **7**. Both **8** and **9** were identified by liquid chromatography‐mass spectrometry (LCMS) analysis of the crude extracts following the feeding (Figure 4B and S13, Supporting Information), providing additional support that AdpST possesses both mono‐ and di‐sulfation enzymatic activity.

Lastly, we expressed recombinant AdpST with an N‐terminal maltose‐binding protein (MBP) tag and a Factor Xa cleavage site in *E. coli* BL21 in order to purify AdpST for in vitro experiments. Following amylose resin chromatography and cleavage of the N‐terminal MBP tag from AdpST, we performed an in vitro assay containing AdpST, PAPS, and **1** (Figure 4C). Incubation for 24 h at 30 °C resulted in the conversion of **1**–**3** and **5**, as observed via MS analysis (Figure S14, Supporting Information). Ultimately, comparison of the results of our heterologous expression, feeding, and in vitro assays (Figure 4D) conclusively confirmed that AdpST was the sole enzyme responsible for both mono‐ and di‐sulfation of adipostatins, making AdpST the first bacterial PAPS‐dependent ST capable of di‐sulfation, including STs from primary metabolism.^[^ ^11^, ^17^, ^20^, ^23^, ^24^, ^25^, ^26^, ^27^, ^28^, ^29^, ^30^ ^]^

### 2.4 The Dedicated PAPS Biosynthetic Cassette Improves Sulfation Capacity

**Figure 5:**
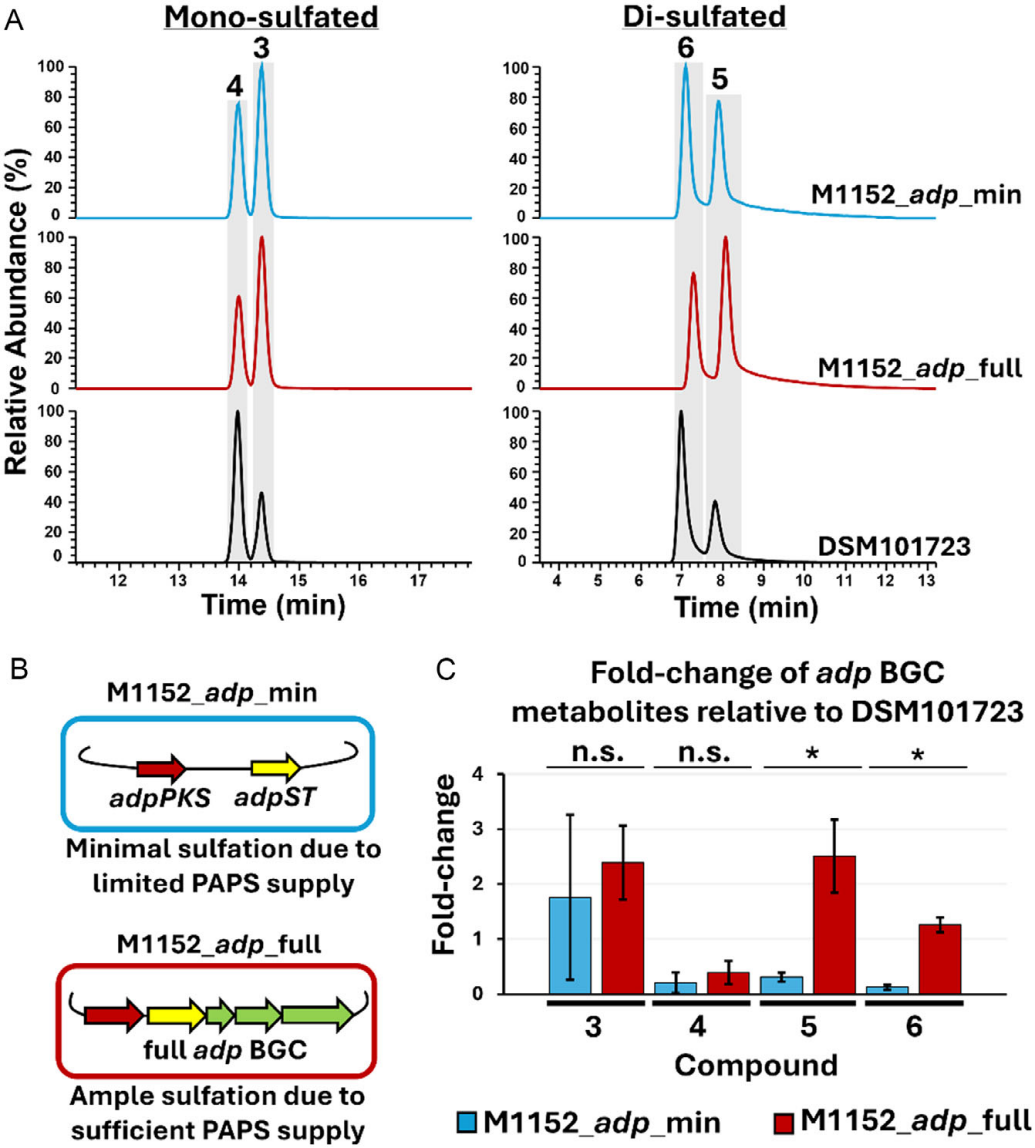
Heterologous expression of *adp*_min and *adp*_full in *S. coelicolor* M1152 results in differential sulfatostatin production. A) EICs of the crude extracts of M1152_*adp*_min, M1152_*adp*_full, and the native *S. davaonensis* DSM101723. EIC range of mono‐sulfated chromatograms is 399.21–399.23; EIC range of di‐sulfated chromatograms is 479.17–479.19. B) Diagram demonstrating the hypothesized effect of the dedicated PAPS biosynthetic cassette on sulfation of adipostatins. C) Fold‐change production of **3–6** in M1152_*adp*_min and M1152_*adp*_full compared to *S. davaonensis* DSM101723, suggesting that the presence of the dedicated PAPS biosynthetic cassette enhances sulfation ability. Fold‐change was determined based on EIC peak area and was normalized against total ion chromatogram intensities (Extended Data File S1, Supporting Information). Cultures were made in biological replicates (*n* = 3), n.s. = not significant, * = statistically significant at *p* < 0.05; error bars are shown representing +/− one standard deviation.

Motivated by the results of our *E. coli* BL21 expression, feeding, and in vitro experiments, we sought to study AdpST in an environment more similar to its native host *S. davaonensis* DSM101723. Thus, we constructed two heterologous expression systems in *Streptomyces coelicolor* M1152, one expressing the minimal genes necessary to produce sulfatostatins, *adpPKS* and *adpST* (M1152_*adp*_min) and the other expressing the full *adp* BGC, including the dedicated PAPS biosynthetic cassette *adpP1*‐*adpP3* (M1152_*adp*_full). Following fermentation of M1152_*adp*_min and M1152_*adp*_full, crude extracts were subjected to MS analysis, along with extracts of the native *S. davaonensis* DSM101723 as a positive control, which revealed the production of **1**–**6** in both M1152_*adp*_min and in M1152_*adp*_full but no production of **7**–**9** (**Figure** **5**A). This confirmed that our *Streptomyces* heterologous hosts more closely simulated the native *S. davaonensis* DSM101723 than the *E. coli* BL21 heterologous host. Furthermore, we sought to compare the sulfatostatin production of M1152_*adp*_min and M1152_*adp*_full, as we hypothesized that the presence of the dedicated PAPS biosynthetic cassette would support greater di‐sulfation activity due to increased levels of PAPS in the cell as the donor for sulfation (Figure 5B). We thus compared the MS peak areas of the sulfated products **3**–**6** in M1152_*adp*_min and M1152_*adp*_full to the peak areas of these compounds from the native *S. davaonensis* DSM101723. This allowed us to both evaluate the impact that the PAPS biosynthetic cassette has on sulfatostatin production and to investigate other factors that may influence mono‐ and di‐sulfation. Our analysis revealed that M1152_*adp*_full produced the mono‐sulfated compounds **3** and **4** at statistically equivalent levels compared to M1152_*adp*_min, but produced greater levels of the di‐sulfated compounds **5** and **6** at *p* < 0.05 (Figure 5C). This supported our hypothesis that the dedicated PAPS biosynthetic cassette plays a major role in the production yield of di‐sulfated adipostatins by increasing the intracellular concentration of PAPS.

### 2.5 AdpST is Phylogenetically Distinct from Characterized Bacterial STs

**Figure 6:**
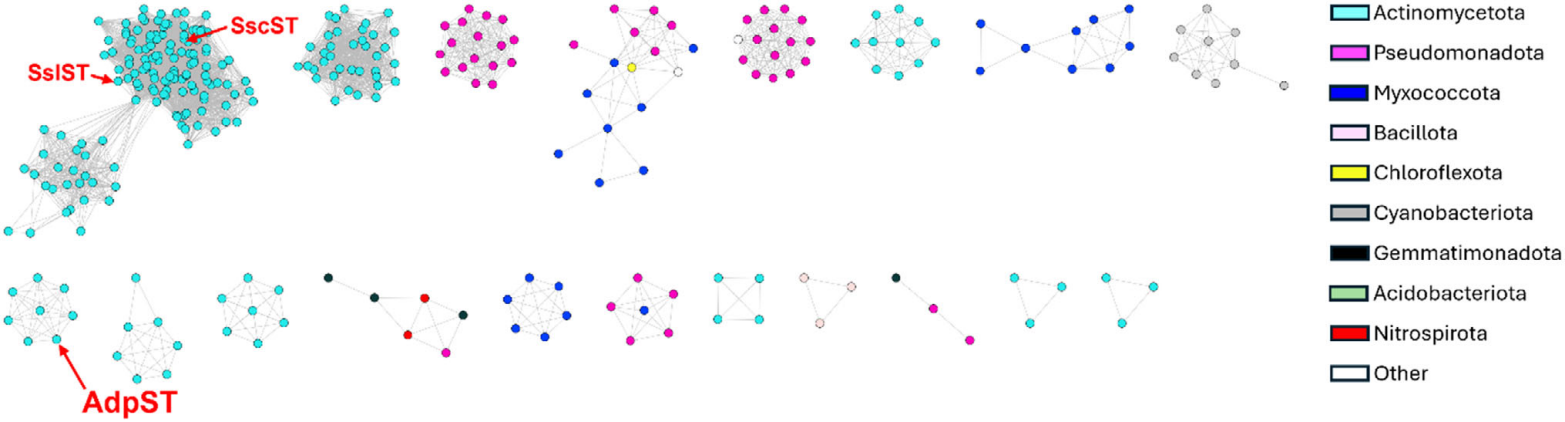
SSN analysis of AdpST against characterized and uncharacterized bacterial STs. AdpST clusters with a group of uncharacterized actinomycete STs, suggesting that other STs may share AdpST's unique di‐sulfation activity. The lanthipeptide STs SscST and SslST clustered with the largest cluster, which contained actinomycete STs. Sequences were analyzed with an *e*‐value of *e* ^−10^.

After confirming the unique status of AdpST as the first bacterial PAPS‐dependent ST capable of di‐sulfation, we sought to investigate the presence of AdpST homologues across microbial phyla by constructing a sequence similarity network (SSN). From this analysis, we found that AdpST clusters with seven uncharacterized STs from actinomycetes that are distinct from characterized STs, including most other actinomycete STs (**Figure** **6**). This suggested that AdpST homologs are indeed present in other actinomycetes, albeit these AdpST homologs seem to be rare compared to the majority of identified actinomycete STs. Intriguingly, we also found that the previously reported lanthipeptide STs SscST and SslST^[^ ^17^ ^]^ clustered with a large group of actinomycete STs distinct from the AdpST cluster (Figure 6). Thus, our SSN analysis demonstrated that AdpST is distinct from characterized STs, while also suggesting that similar STs to AdpST exist in nature, which may perform similarly unique sulfation reactions to generate chemically novel metabolites.

**Figure 7:**
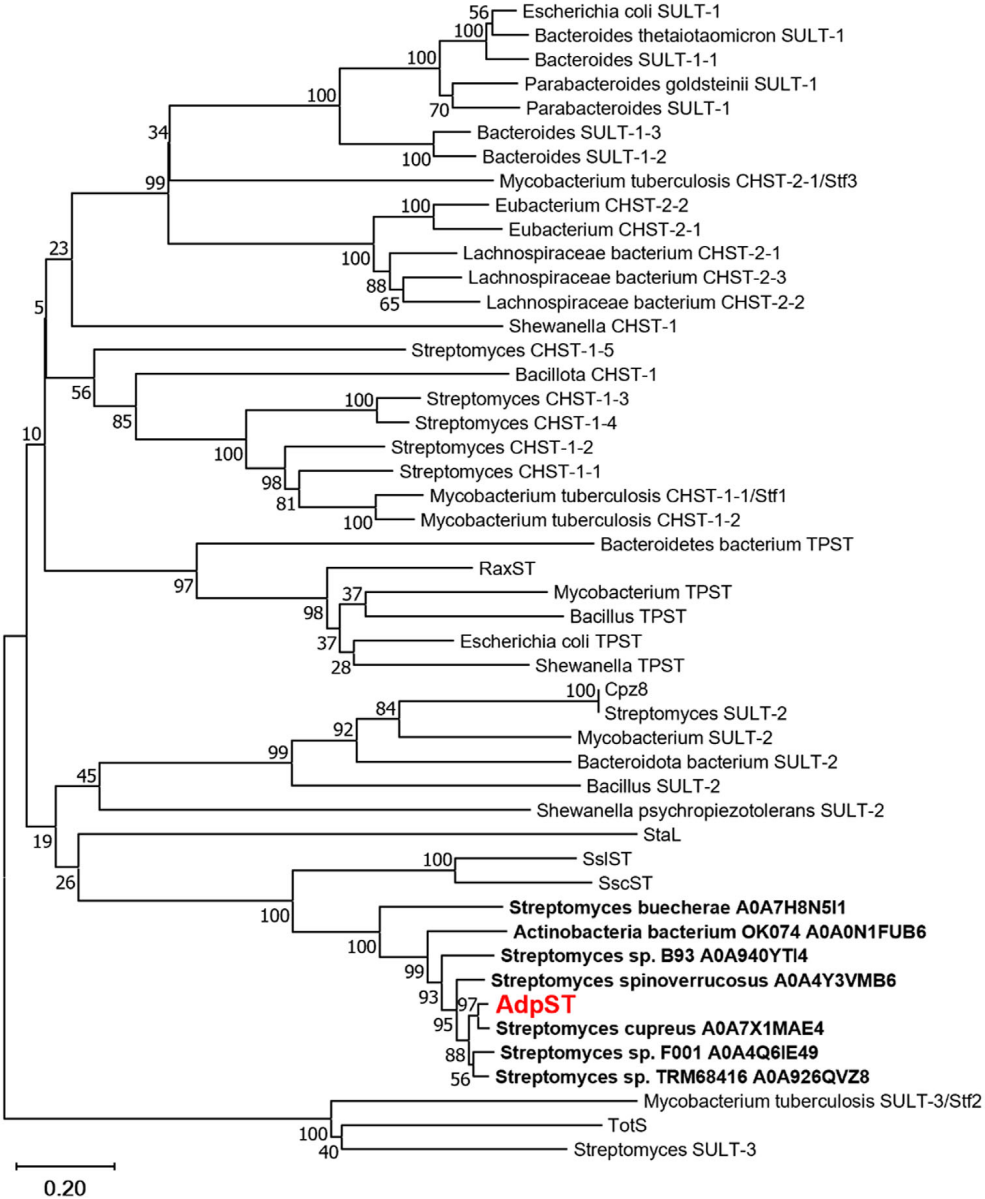
Phylogenetic analysis of AdpST and 47 bacterial STs demonstrates that AdpST is phylogenetically distant from most known bacterial STs but is related to seven uncharacterized actinomycete STs identified in SSN analysis and to the lanthipeptide STs SslST and SscST. In the tree, the ST sequences identified from SSN analysis are bolded and are annotated with the species name from which they originate and the UniProt accession code.

After identifying several AdpST homologs in our SSN analysis, we sought to determine the phylogenetic relationship of AdpST to the SSN‐identified homologues and to 40 previously reported bacterial STs, including the six secondary metabolite STs previously discussed^[^ ^11^, ^17^, ^19^, ^20^, ^24^ ^]^ and 34 STs from a recent evolutionary study,^[^ ^31^ ^]^ and thus we subsequently constructed a phylogenetic tree. Expectedly, the phylogenetic analysis demonstrated AdpST to be closely related to the STs identified in our SSN analysis. However, AdpST diverged from most of the remaining STs in our phylogenetic analysis, suggesting little homology to these known STs (**Figure** **7**). Intriguingly, the most closely related STs to AdpST besides the SSN‐identified STs were SscST and SslST, the STs involved in the biosynthesis of sulfotyrosine‐containing lanthipeptides, potentially suggesting that AdpST, SslST, and SscST evolved from a common ancestor. This close relationship is further supported by the presence of dedicated PAPS biosynthetic cassettes in the *ssl*, *ssc*, and *adp* BGCs. Despite their close phylogenetic relationship to AdpST, however, SslST and SscST were not reported to exhibit di‐sulfation activity. Thus, it is likely that these STs evolved from a common ancestor but diverged in their substrate selectivity and enzymatic activity. Collectively, our SSN and phylogenetic tree analyses suggested that AdpST is distantly related to most known bacterial STs but shares homology with a small group of uncharacterized STs.

## 3 Conclusions

Here, we have identified new analogs of adipostatins from *S. davaonensis* DSM101723 with mono‐ or di‐sulfated resorcinol head groups. We investigated the novel ST involved in the biosynthesis of these adipostatins, AdpST, and have used heterologous expression, feeding, and in vitro assays to demonstrate that AdpST exhibits di‐sulfation activity, which is unprecedented for bacterial PAPS‐dependent STs. We further showed that the *adp* BGC, which encodes AdpST, also contains a PAPS biosynthetic cassette, and demonstrated that this dedicated biosynthetic cassette ensures ample sulfate donor substrate is available for di‐sulfation of adipostatins. Furthermore, our phylogenetic analysis showed that AdpST diverges from most known bacterial STs. Lastly, our SSN analysis revealed that AdpST clusters with a small group of uncharacterized actinomycete STs, suggesting that additional AdpST‐like STs exist in other organisms and may have unique di‐sulfation activity.

## 4 Experimental Section

4.1

4.1.1

##### General Materials

*S. davaonensis* DSM101723 was purchased from The Leibniz Institute DSMZ. LCMS and HPLC solvents were purchased from Fisher Scientific. Chemical reagents, biochemicals, and media components used in this study were purchased from Thermo Fisher Scientific Co. Ltd. (USA) unless otherwise stated. Restriction endonucleases were purchased from New England Biolabs, Inc. (USA). PrimeSTAR HS DNA polymerase (Takara Biotechnology Co., Ltd. Japan) was used for all polymerase chain reaction (PCR) amplifications on an Eppendorf Mastercycler Nexus X2 Thermal Cycler (Eppendorf Co., Ltd. Germany). PCR products were purified using the E.Z.N.A. Gel Extraction Kit (Omega Bio‐tek, Inc., USA). The NEBuilder HiFi DNA Assembly master mix (New England Biolabs, Inc., USA) was applied for Gibson assembly. All plasmids were extracted using the E.Z.N.A. Plasmid DNA Mini Kit I (Omega Bio‐tek, Inc., USA). Oligonucleotide synthesis was performed by Eton Bioscience, Inc. (USA).

##### Bioinformatic Analysis

BGCs in the *S. davaonensis* DSM101723 genome were identified using antiSMASH 7.1.0 with relaxed (default) detection strictness and all extra features on. The results were manually analyzed to identify the *adp* BGC.

##### Identification and Isolation of Sulfated Adipostatins from S. Davaonensis DSM101723

The native strain *S. davaonensis* DSM101723 was recovered on mannitol salt (MS) agar after incubating at 30 °C for 7 days. A single colony was picked and transferred to 5 mL tryptic soy broth (TSB) which was pre‐cultured overnight at 30 °C with shaking. The pre‐culture was subsequently inoculated (1% v/v) into 25 mL sporulation medium (SPM) and allowed to ferment at 30 °C for 8 days with shaking. The fermented culture was then pelleted by centrifugation at 3186 × g, and the pellet and supernatant were extracted separately by ethyl acetate.

##### Liquid Chromatography, High‐Resolution Mass Spectrometry, and Tandem Mass Spectrometry

High‐resolution mass spectra were obtained following high‐performance liquid chromatography on a Vanquish UHPLC with solvent A (60/40 ACN/H_2_O + 0.1% formic acid) and solvent B (90/10 IPA/ACN + 0.1% formic acid). A gradient solvent composition was used at 0.2 mL min^−1^, beginning by maintaining 95% A/5% B from 0–3 min, then increasing from 95% A/5% B to 2% A/98% B from 3–22 min, then maintaining 2% A/98% B from 22–32 min, then returning from 2% A/98% B to 95% A/5% B from 32–33 min. High‐resolution mass spectra were obtained with electrospray ionization on a Q‐Exactive OrbiTrap operating in negative ion mode at 60,000 resolution; high‐resolution tandem mass spectra were collected on the same instrument at 30,000 resolution.

Low‐resolution mass spectra (collected for experiments in the “Heterologous expression, feeding, and in vitro assays” section below) were collected on a Thermo Fisher LTQ XL Linear Ion Trap Mass Spectrometer interfaced with a Thermo Fisher Ultimate 3000 UHPLC. UHPLC conditions for all low‐resolution mass spectra were as follows for solvent A (acetonitrile + 0.1% formic acid) and solvent B (H_2_O + 0.1% formic acid): 10% A/90% B to 100% A at 0 min, increasing linearly to 100% A at 10 min, followed by maintaining 100% A until 12.5 min, then returning linearly to 10% A/90% B at 15 min.

##### NMR Structural Characterization

NMR spectra were obtained on a Bruker Avance III HD 500 MHz spectrometer with a PA BBO 500S2 BBF‐H‐D_05 Z SP probe. The CD_3_OD and D_2_O used for NMR experiments were purchased from ACROS Organics.

##### Heterologous Expression, Feeding, and In Vitro Assays

*S. davaonensis* DSM101723 genomic DNA was extracted using standard protocols. All oligonucleotide primers used for PCR were designed using Snapgene software and synthesized by Eton Bioscience Inc. These primers were used in PCR reactions to amplify *adpPKS* and *adpST* from the *S. davaonensis* DSM101723 genome. The amplified *adpPKS* segment was then assembled into a pACYC vector following linearization with appropriate restriction enzymes, and the *adpST* segment was assembled into a linearized pHis8 vector via Gibson Assembly. The assembled plasmids pACYC_*adpPKS* and pHis8_*adpST* were then successively transformed into *E. coli* BL21 to yield BL21_*adpPKS*_*adpST*. For expression of **7–9**, BL21_*adpPKS*_*adpST* was pre‐cultured overnight in terrific broth (TB) supplemented with kanamycin and chloramphenicol to maintain selection pressure for pACYC_*adpPKS* and pHis8_*adpST*, respectively. The pre‐culture was then inoculated (1%) into 30 mL of TB supplemented with kanamycin and chloramphenicol and grown at 37 °C to OD600 = ≈0.7, at which point 0.2 mm isopropyl ß‐D‐1‐thiogalactopyranoside (IPTG) was added to the culture to induce *adpPKS* and *adpST* expression. The culture temperature was then decreased to 16 °C for 16 h, after which the cells were pelleted and the pellet was extracted using butanol at a volume equal to the original culture volume. After drying under nitrogen, the extract was redissolved in methanol, and production of **7–9** was checked via LCMS analysis.

For feeding assays, an *E. coli* BL21 strain was first constructed by amplifying *adpST* from *S. davaonensis* DSM101723 genomic DNA using appropriate primers and assembling the amplified segment with a linearized pMAL vector. This pMAL_*adpST* plasmid was then transformed into *E. coli* BL21 and an overnight culture of this BL21_*adpST* was started in lysogeny broth (LB) supplemented with ampicillin. The pre‐culture was then inoculated (1%) into a 30 mL culture of TB supplemented with ampicillin and grown at 37 °C to OD600 = ≈0.7, at which point compound **7** was added to the culture (0.2 mm) along with 0.2 mm IPTG to induce *adpST* expression. The culture temperature was then decreased to 16 °C for 16 h, after which the cells were pelleted and the pellet was extracted with butanol. After drying the extract under nitrogen, the extract was redissolved in methanol and production of **8** and **9** was checked via LCMS analysis.

For in vitro assays, *adpST* was amplified from *S. davaonensis* DSM101723 genomic DNA using appropriate primers and then assembled with a linearized pMAL vector to add an MBP tag at the N‐terminus of AdpST with a Factor Xa cleavage site. Subsequently, pMAL_MBP_*adpST* was transformed into *E. coli* Rosetta and an overnight pre‐culture in LB supplemented with ampicillin and chloramphenicol was made. The overnight pre‐culture was then inoculated (1%) into 500 mL TB supplemented with ampicillin and chloramphenicol and grown at 37 °C until OD600 = ≈0.7, at which point 0.2 mm IPTG was added to induce *adpST* expression. The culture temperature was then decreased to 16 °C for 16 h, after which the cells were pelleted and the supernatant was removed. After resuspension in lysis buffer (20 mm Tris, pH 8.0, 300 mm NaCl, 20 mm imidazole, and 5% glycerol), the lysate was added to an amylose resin column and was finally eluted with 10 mm maltose. Following purification, the MBP tag was cleaved from MBP_AdpST by addition of Factor Xa and incubation at 20 ºC for 24 h. Following purification of AdpST from the Factor Xa reaction mixture, 20 μm of AdpST was added into a 1.5 mL microcentrifuge tube containing 50 mm (2‐(N‐morpholino)ethanesulfonic acid in ddH_2_O at pH = 6.5, 5 mm MgCl_2_, 200 μm PAPS, and 200 μm compound **1** in a total reaction volume of 50 μL. The reaction mixture was then incubated at 30 °C for 24 h, after which the reaction products were extracted via butanol, dried under N_2_, and redissolved in methanol prior to MS analysis.

For expression in *S. coelicolor* M1152, appropriate primers were used to PCR amplify the minimal *adp* BGC (*adpPKS* and *adpST,* amplified separately) and the entire *adp* BGC (including *adpP1‐adpP3*, amplified whole BGC). The amplified *adpPKS* was assembled into a pCAP01 vector, *adpST* was assembled into a pKY01 vector, and the whole *adp* BGC was assembled into a separate pCAP01 vector using Gibson Assembly. The pCAP01 and pKY01 vectors encode a phage φC31 integrase and contain an attP site for chromosomal integration in *Streptomyces*. Following assembly of pCAP01_*adpPKS*, pKY01_*adpST*, and pCAP01_*adp*_full, each plasmid was transformed into *E. coli* DH10B for maintenance of the plasmids, which were subsequently extracted through standard protocols. Each plasmid was then transformed into separate *E. coli* ET12567 strains, which were then used to conjugate the plasmids into their *S. coelicolor* M1152 hosts via triparental mating with *E. coli* ET12567_pUB307, *E. coli* ET12567 containing the respective pCAP01 plasmid, and *S. coelicolor* M1152. For M1152_*adp*_min conjugation, pCAP01_*adpPKS* was conjugated; first after successful conjugation, pKY01_*adpST* was then conjugated to the same strain to yield M1152_*adp*_min. For M1152_*adp*_full, pCAP01_*adp*_full was conjugated into *S. coelicolor* M1152.

After construction of the *S. coelicolor* M1152 heterologous hosts, M1152_*adp*_min and M1152_*adp*_full, and *S. davaonensis* DSM101723 was each pre‐cultured at 30 °C for 3 days in 10 mL of TSB supplemented with appropriate antibiotics depending on the plasmid present in the heterologous expression strain (kanamycin for pCAP01 plasmids and apramycin for pKY01 plasmids). Each pre‐culture was then inoculated (1%) into 30 mL of SPM without antibiotics and cultured at 30 °C for 8 days. The cells were then pelleted by centrifugation and the pellet was extracted with 10 mL ethyl acetate. The extracts were dried under nitrogen and redissolved in equal volumes of methanol (200 μL). Subsequently, 0.2 μL of each sample was injected during LCMS analysis.

Following LCMS analysis, the peak areas for each peak in the M1152_*adp*_min, M1152_*adp*_full, and *S. davaonensis* DSM101723 samples were obtained using the following EIC *m*/*z* ranges: 399.21–399.23 for compound **3** and compound **4**; 479.17–479.19 for compound **5** and compound **6**. For compound **3**, the EIC peak area for **3** in each M1152_*adp*_min sample was obtained and was then divided by the TIC peak area for the respective sample, yielding the EIC/TIC value (normalization step).^[^ ^32^, ^33^ ^]^ This process was repeated for the remaining compounds **4–6** in the M1152_*adp*_min samples and for compounds **3–6** in the M1152_*adp*_full and *S. davaonensis* DSM101723 samples. The EIC/TIC samples for the *S. davaonensis* DSM101723 samples were then averaged to allow for fold‐change calculation. The EIC/TIC values for compound **3** from the M1152_*adp*_min samples were then divided by the average EIC/TIC value of the *S. davaonensis* DSM101723 samples, yielding the normalized fold‐change of **3** for M1152_*adp*_min. The three normalized fold‐change values were then averaged together. This process was repeated for the remaining compounds **4–6** from the M1152_*adp*_min samples and for compounds **3–6** from M1152_*adp*_full. Standard deviations were then obtained for the normalized fold‐change values, along with p values from two‐tailed *t*‐tests assuming unequal variance; these values were then used to construct the plot shown in Figure 5C.

##### Phylogenetic and Sequence Similarity Analyses

The AdpST SSN was generated using the Enzyme Function Initiative‐Enzyme Similarity Tool (EFI‐EST).^[^ ^34^ ^]^ The parameters used to identify sequences were an e‐value of 10, 10,000 maximum sequences retrieved, using the UniProt sequence database, with the taxonomy filter set to “Bacteria, Archaea, and Fungi”.

For the phylogenetic tree construction, evolutionary history was inferred using the Neighbor‐Joining method.^[^ ^35^ ^]^ The optimal tree is shown. The percentage of replicate trees in which the associated taxa clustered together in the bootstrap test (500 replicates) are shown below the branches.^[^ ^36^ ^]^ The tree is drawn to scale, with branch lengths in the same units as those of the evolutionary distances used to infer the phylogenetic tree. The evolutionary distances were computed using the Poisson correction method^[^ ^37^ ^]^ and are in the units of the number of amino acid substitutions per site. This analysis involved a total of 48 amino acid sequences. All ambiguous positions were removed for each sequence pair (pairwise deletion option). Evolutionary analyses were conducted in MEGA11.^[^ ^38^ ^]^

## Conflict of Interest

The authors declare no conflict of interest.

## Supporting information

Supplementary Material
