# Supplementary material for: A Bacterial Sulfotransferase Catalyzes an Unusual Di‐Sulfation in Natural Products Biosynthesis
Source: Chembiochem. 2025 Apr 4;26(11):e202500024. doi: 10.1002/cbic.202500024 (PMC12135136; doi:10.1002/cbic.202500024)
Supplement: Supplementary file 1 — Supplementary Material [file CBIC-26-e202500024-s001.zip › cbic202500024-sup-0002-SuppData-S1/cbic202500024-sup-0001-SuppData-S1.pdf]

Supporting Information

A Bacterial Sulfotransferase Catalyzes An Unusual Di-sulfation in Natural Products Biosynthesis

Conor Pulliam+[a], Lukuan Hou+[a], Dan Xue[a], Mingming Xu[a], Katherine Holandez-Lopez[a], Jie Li[a]*

[a] Conor Pulliam, Lukuan Hou, Dan Xue, Mingming Xu, Katherine Holandez-Lopez - Department of Chemistry and Biochemistry, University of South Carolina, Columbia, South Carolina 29208, United States.

Jie Li* - Department of Chemistry and Biochemistry, University of South Carolina, Columbia, South Carolina 29208, United States; orcid.org/0000-0001-7977-6749; Email: LI439@mailbox.sc.edu.

+These authors contributed equally

*Corresponding author

Table of Contents

Supplementary Information Figures S3
Figure S1: High resolution MS analysis of 1 and 2 .... S3
Figure S2: High resolution MS analysis of 3 and 4 .... S4
Figure S3: High resolution MS analysis of 5 and 6 .... S5
Figure S4: ^{1}H-NMR (500 MHz, CD_3OD) spectrum of 1 .... S6
Figure S5. ^{13}C-NMR (125 MHz, CD_3OD) spectrum of 1 .... S7
Figure S6. ^{1}H-NMR (500 MHz, CD_3OD) spectrum of 2 .... S8
Figure S7. ^{13}C-NMR (125 MHz, CD_3OD) spectrum of 2 .... S9
Figure S8. ^{1}H-NMR (500 MHz, D_2O) spectrum of 6 .... S10
Figure S9. ^{13}C-NMR (125 MHz, D_2O) spectrum of 6 .... S11
Figure S10. Mass spectrum of 7–9 produced by BL21_*adpPKS_adpST* S12
Figure S11. ^{1}H-NMR (500 MHz, CD_3OD) spectrum of 7 .... S13
Figure S12. ^{13}C-NMR (125 MHz, CD_3OD) spectrum of 7 .... S14
Figure S13. MS analysis of BL21_*adpST* feeding assay S15
Figure S14. MS analysis of AdpST *in vitro* enzymatic assay S16

Supplementary Information Tables S17
Table S1: NMR data for compound 1 in CD_3OD S17
Table S2: NMR data for compound 2 in CD_3OD S18
Table S3: NMR data for compound 6 in D_2O S19
Table S4: NMR data for compound 7 in CD_3OD S20
Table S5: Primers used in this study S21

Figure S1. High resolution MS analysis of **1** and **2** produced by *S. davaonensis* DSM101723. The chromatogram shown is an EIC with a range of 319.25-319.27.

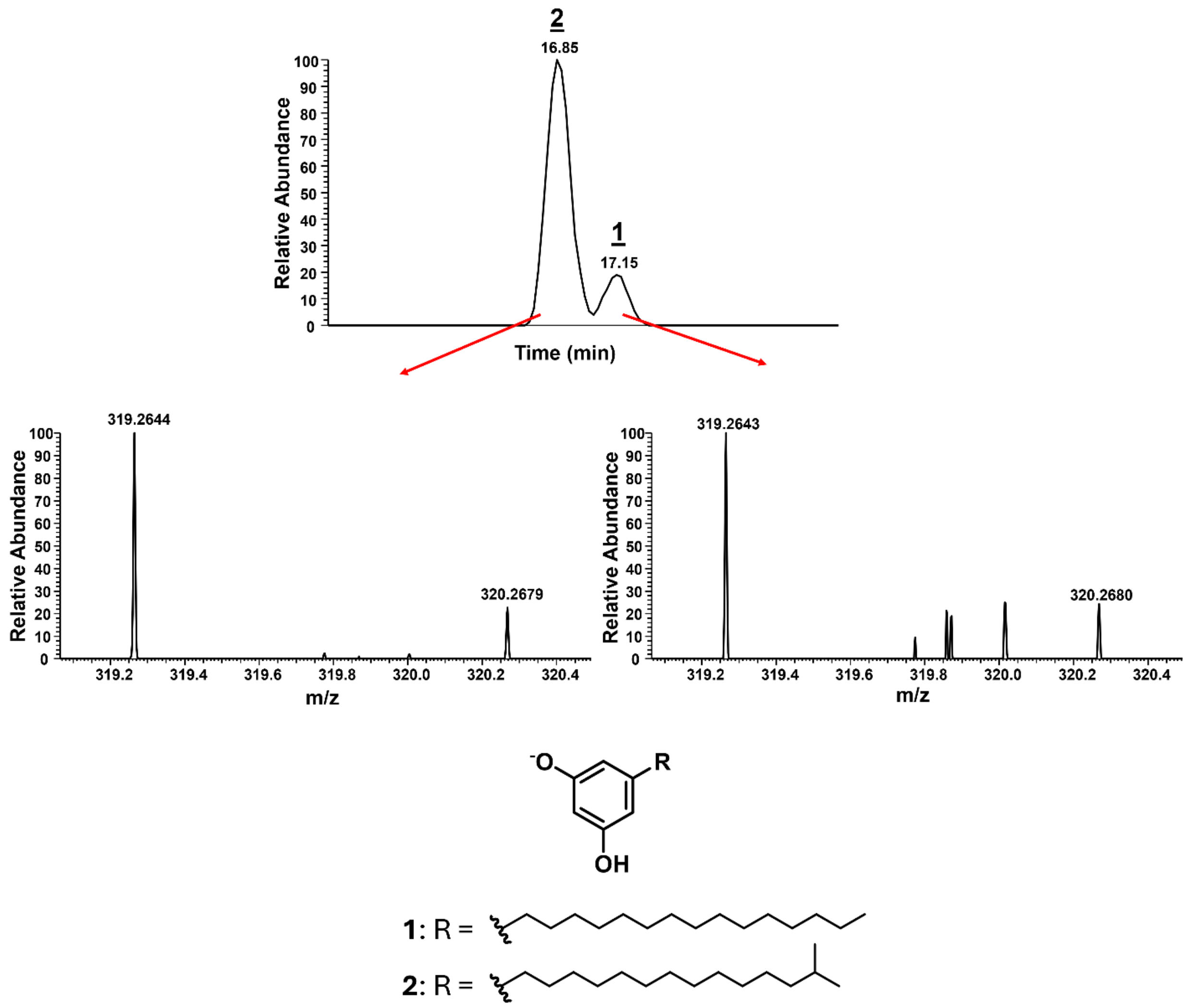

Figure S2. High resolution MS analysis of **3** and **4** produced by *S. davaonensis* DSM101723. The chromatogram shown is an EIC with a range of 399.21-399.23.

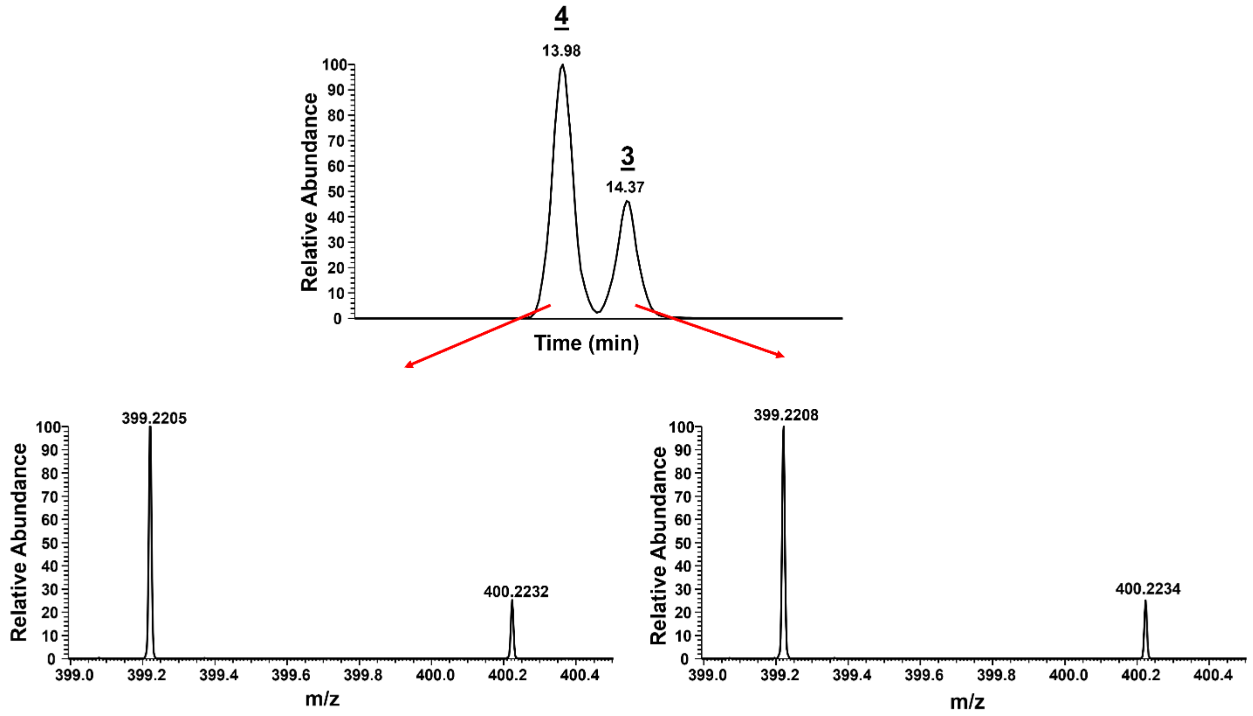

Figure S3. High resolution MS analysis of **5** and **6** produced by *S. davaonensis* DSM101723. The chromatogram shown is an EIC with a range of 479.17-479.19.

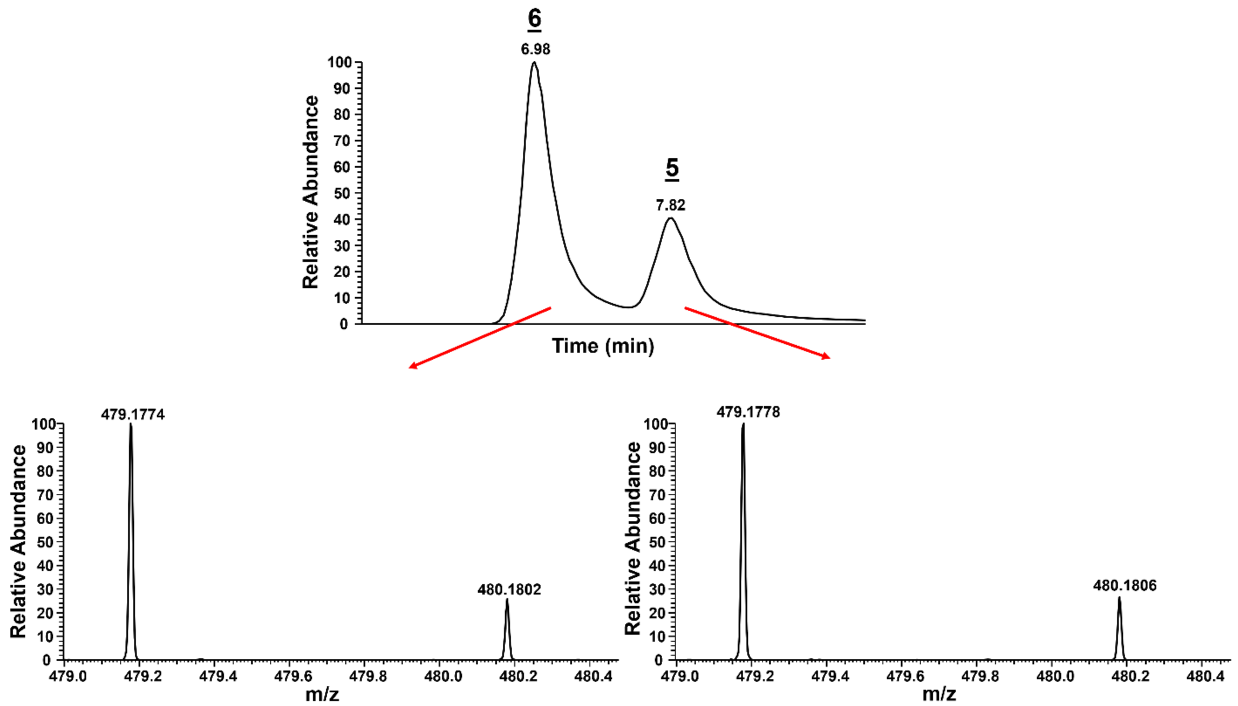

Figure S4. ^{1}H-NMR (500 MHz, CD_3OD) spectrum of **1**.

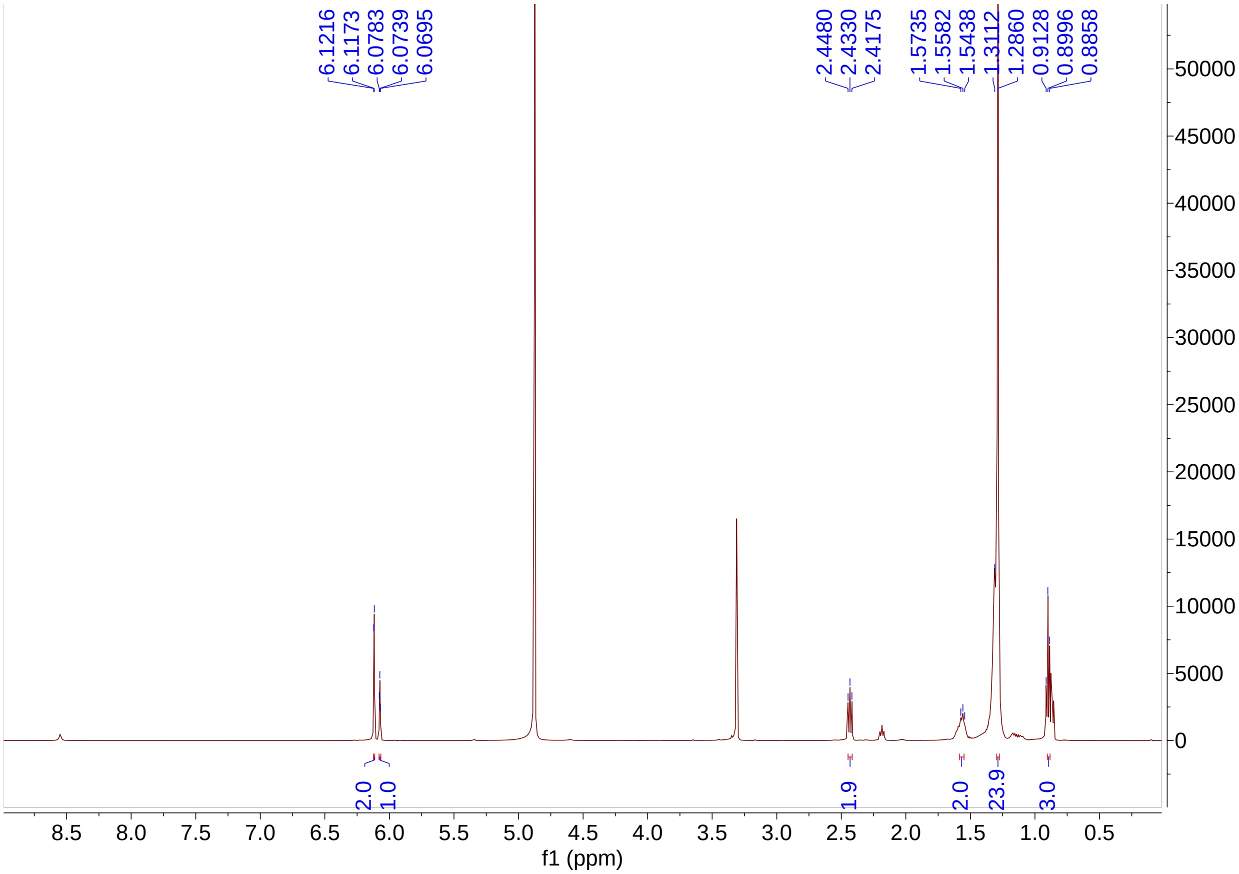

Figure S5. ^{13}C-NMR (125 MHz, CD_3OD) spectrum of **1**.

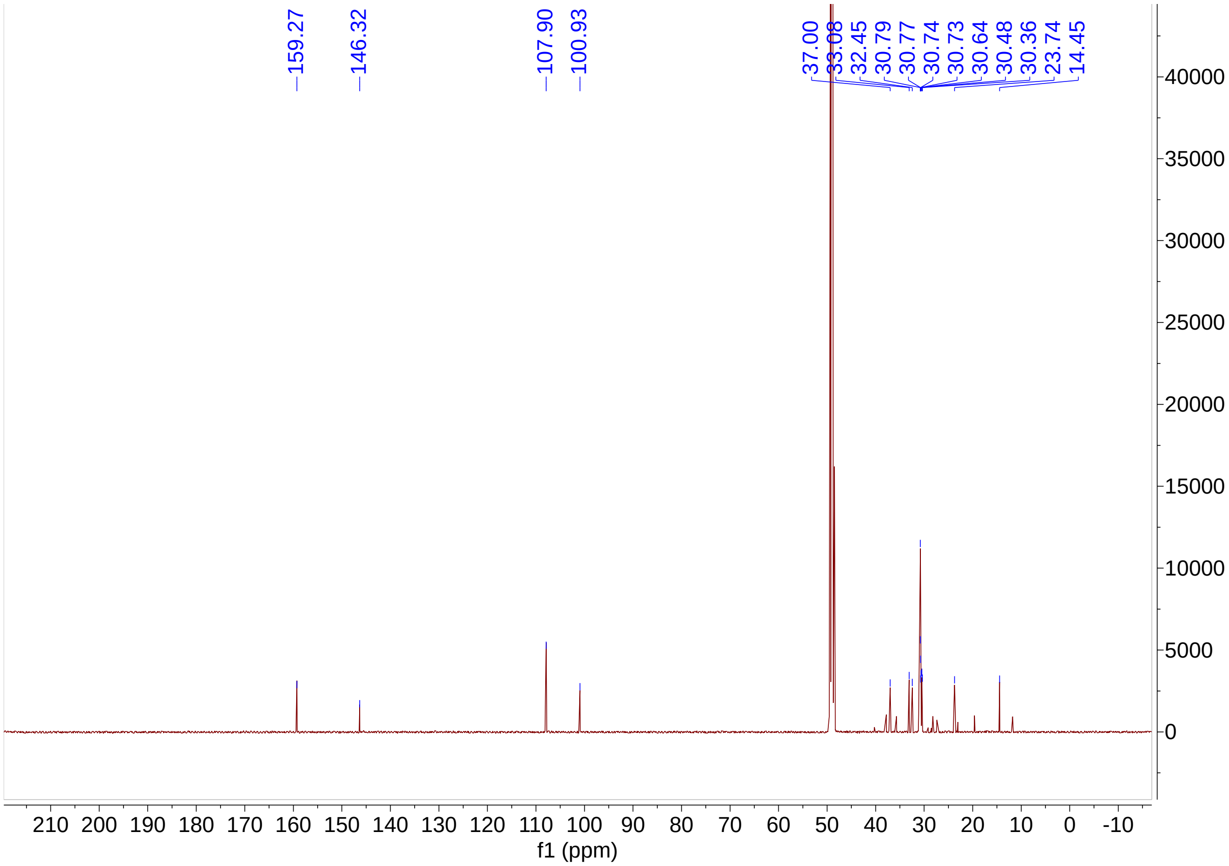

Figure S6. ^{1}H-NMR (500 MHz, CD_3OD) spectrum of **2**.

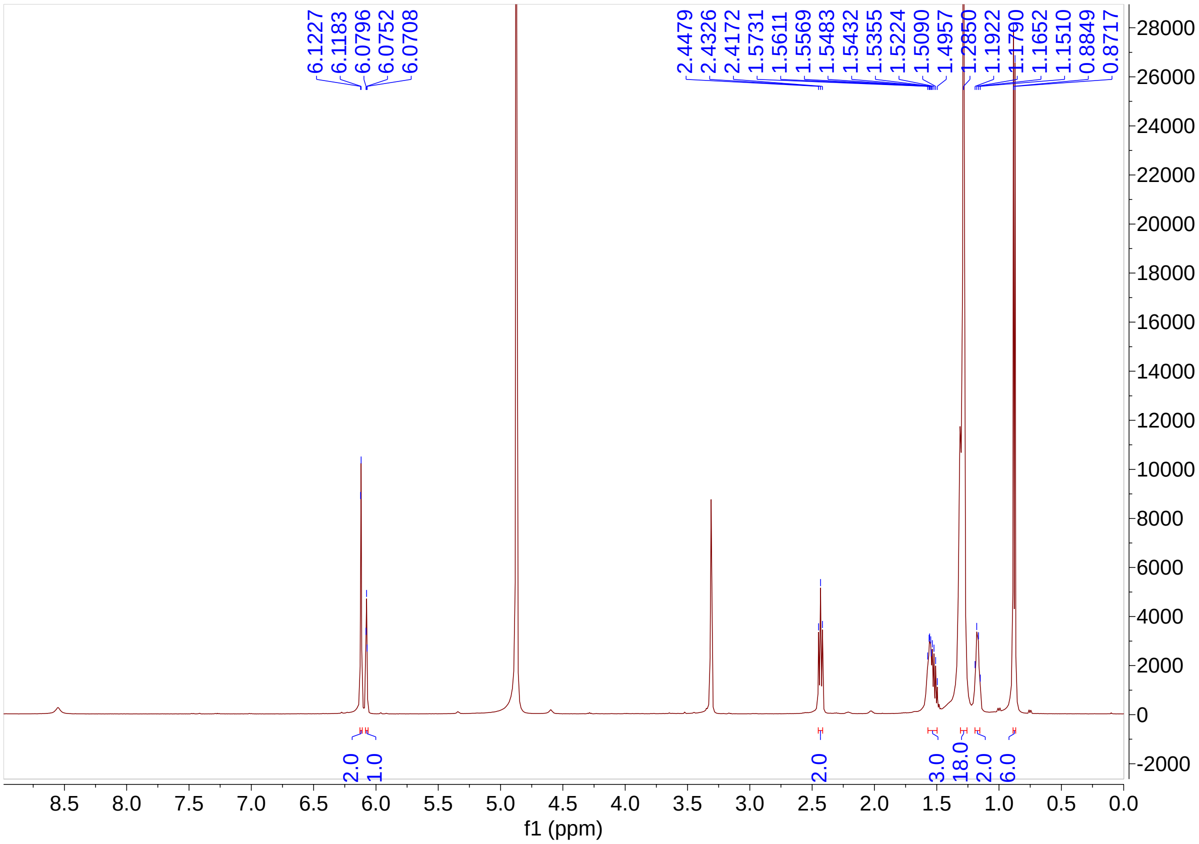

Figure S7. ^{13}C-NMR (125 MHz, CD_3OD) spectrum of **2**.

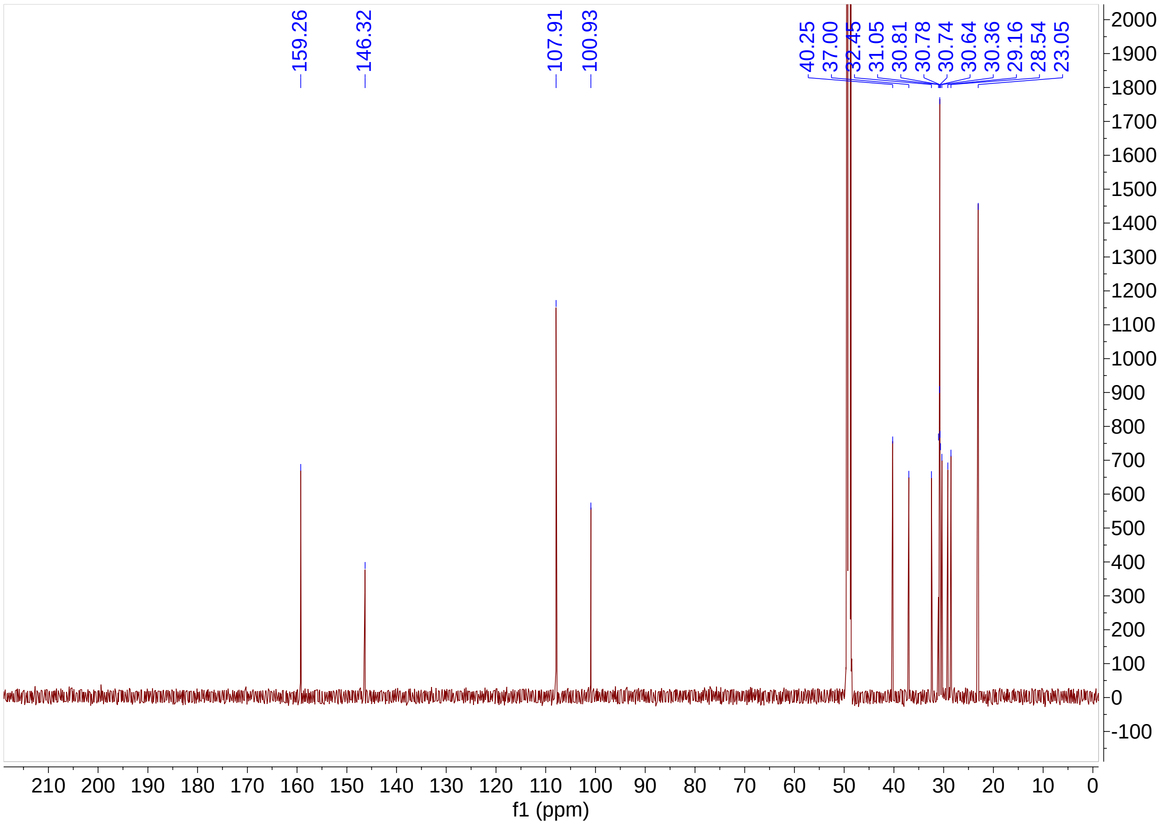

Figure S8. ^{1}H-NMR (500 MHz, D_2O) spectrum of **6**.

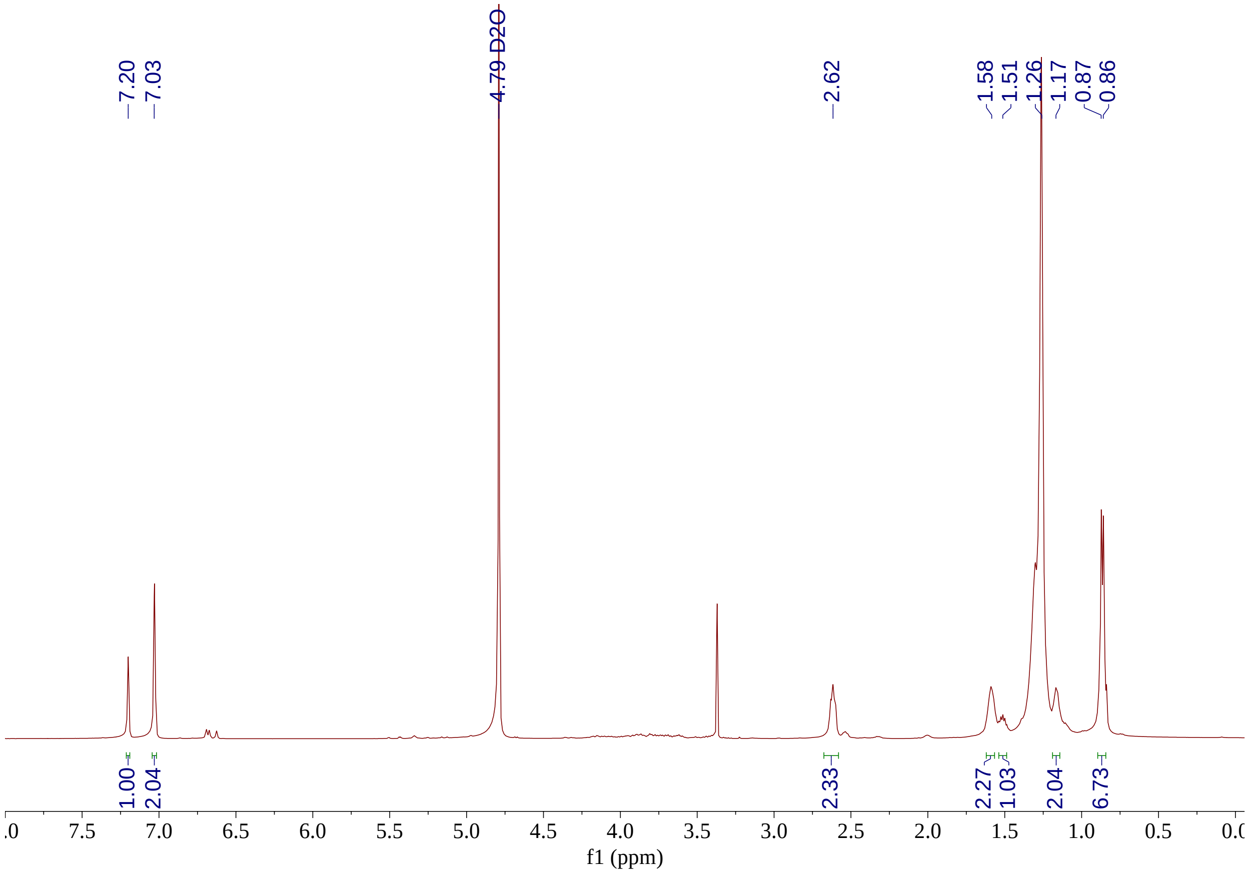

Figure S9. ^{13}C-NMR (125 MHz, D_2O) spectrum of **6**.

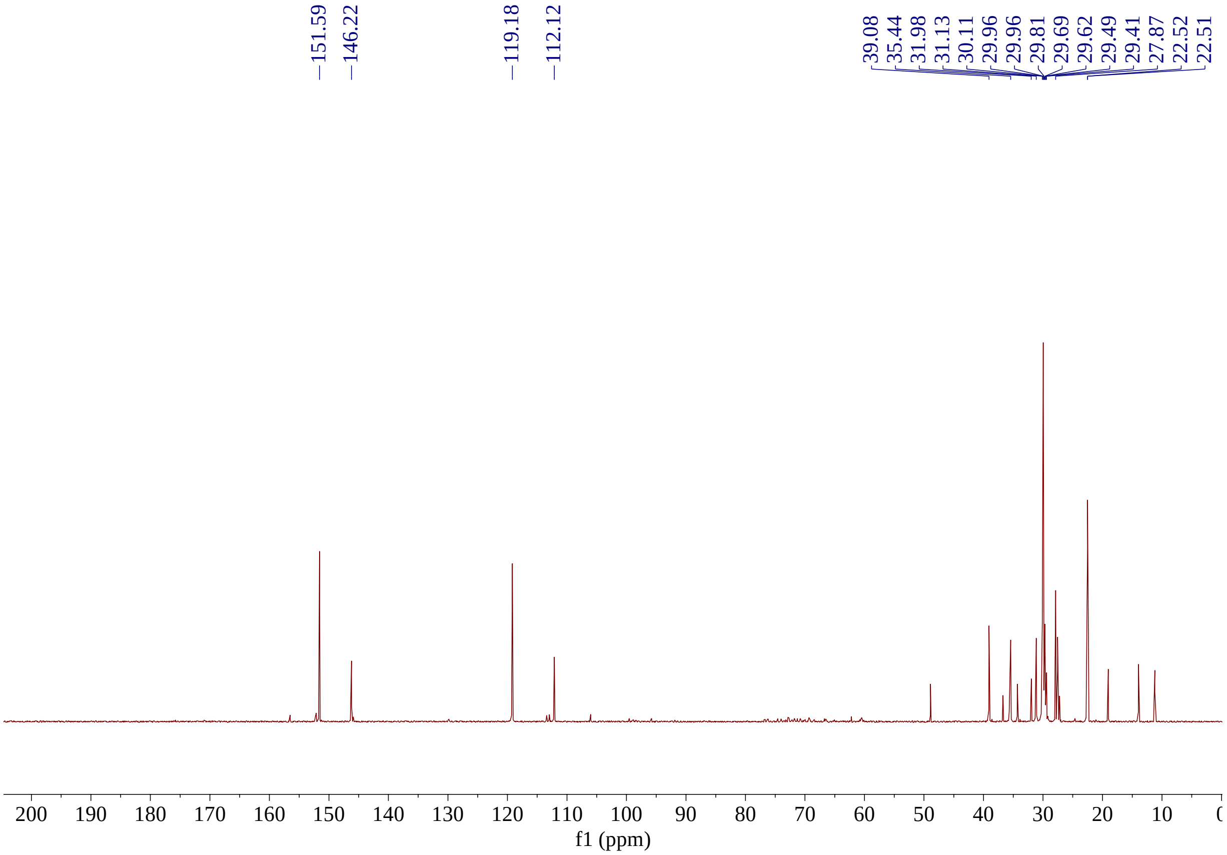

Figure S10. Mass spectrum of **7**–**9** produced by BL21_*adpPKS*_*adpST*.

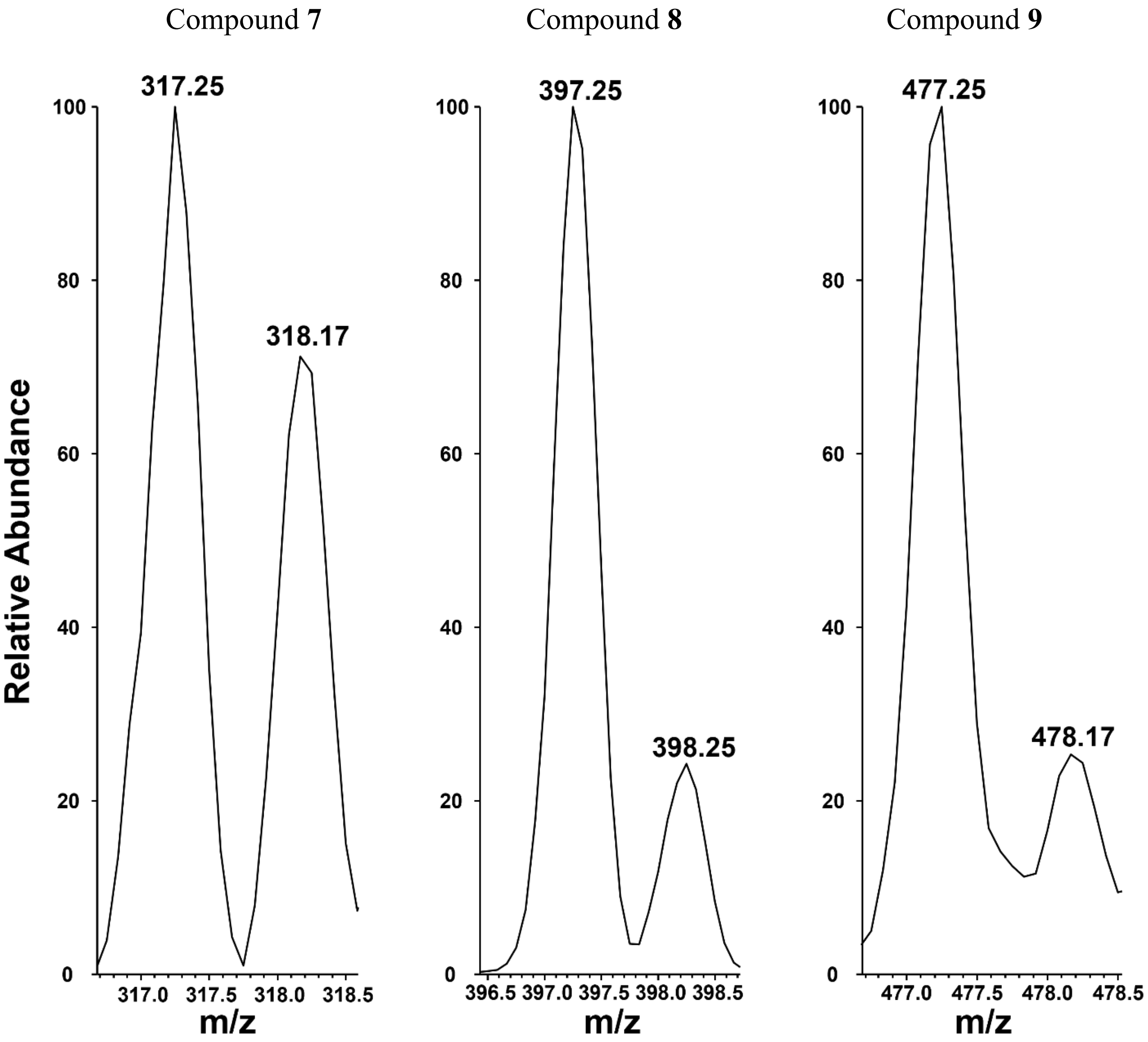

Figure S11. ^{1}H-NMR (500 MHz, CD_3OD) spectrum of **7**.

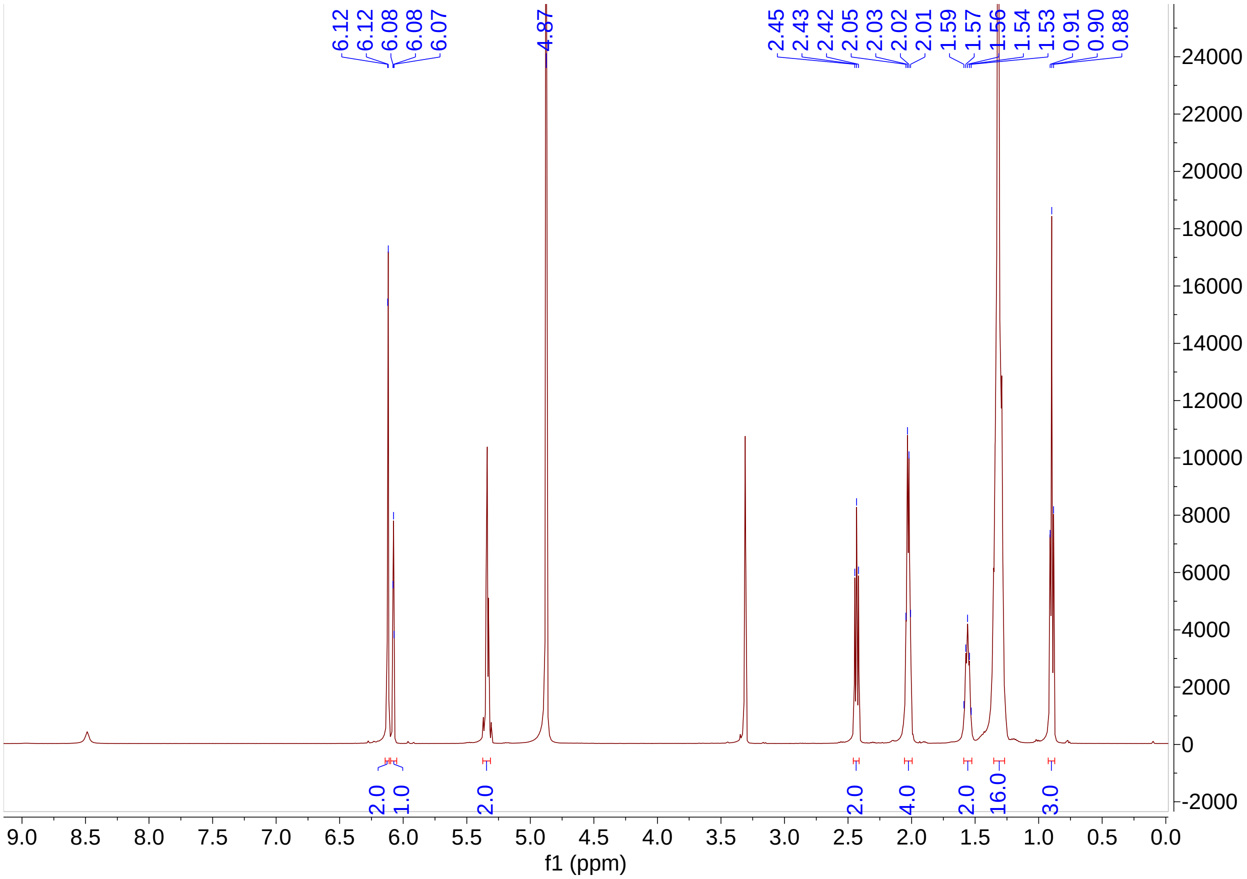

Figure S12. ^{13}C-NMR (125 MHz, CD_3OD) spectrum of **7**.

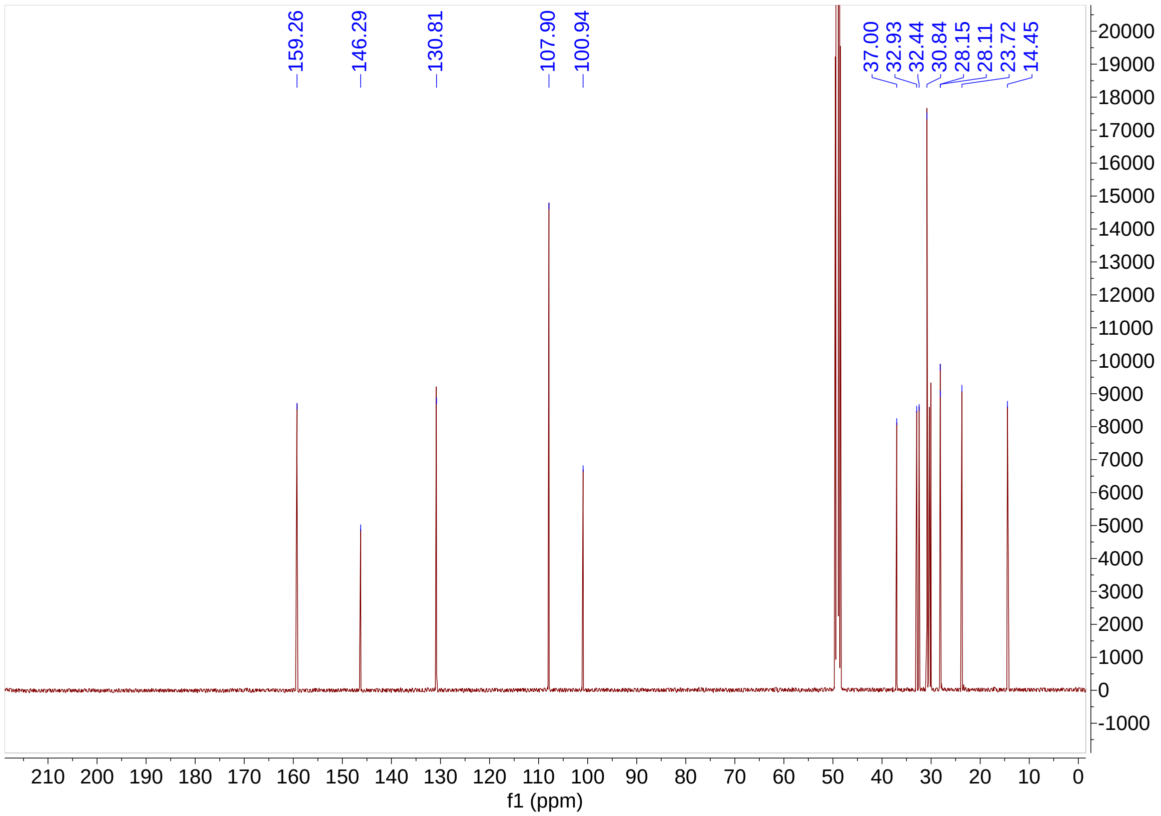

Figure S13. MS analysis of BL21_*adpST* feeding assay. The chromatogram shown for compound **8** is an EIC with a range of 396.5-397.5. The chromatogram shown for compound **9** is an EIC with a range of 476.5-477.5.

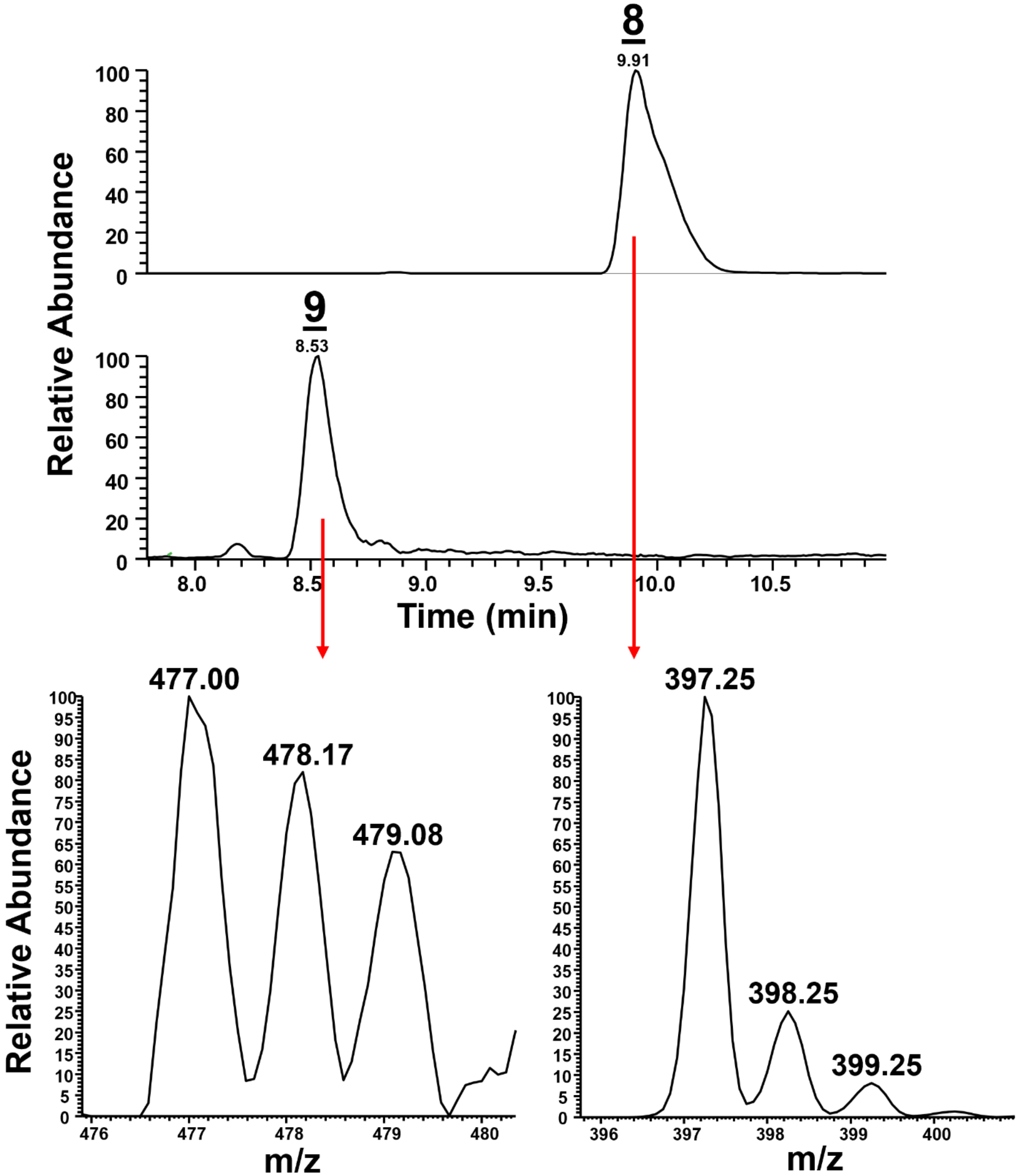

Figure S14. MS analysis of AdpST *in vitro* enzymatic assay. The chromatogram shown for compound **3** is an EIC with a range of 398.5-399.5. The chromatogram shown for compound **5** is an EIC with a range of 478.5-479.5.

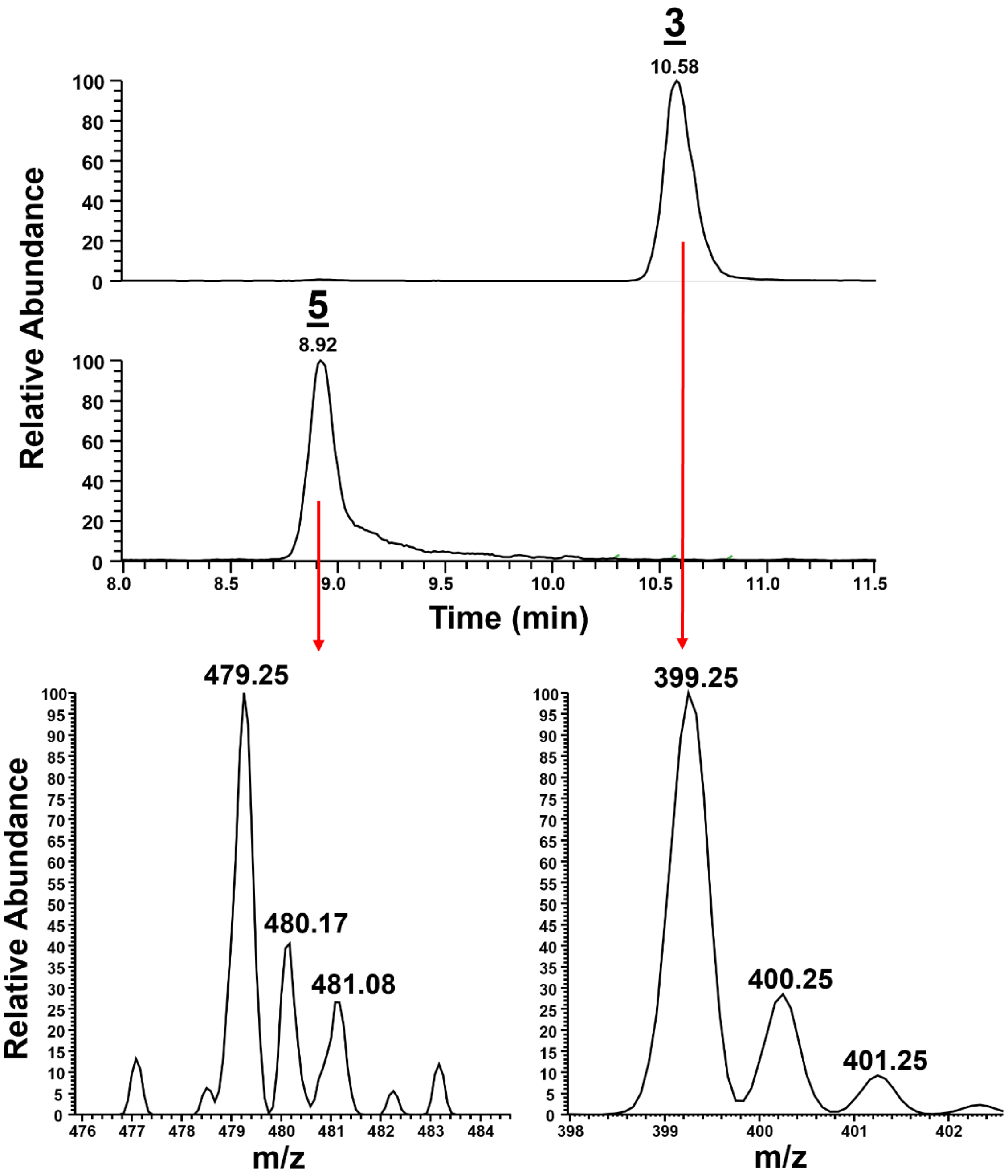

Table S1. NMR resonance assignments for compound **1** in CD_3OD.

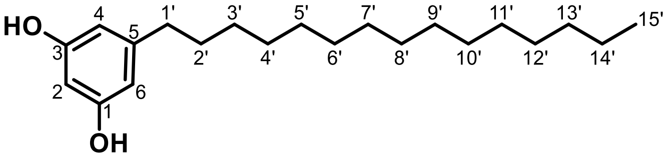

Number	^{1}H-NMR (500 MHz)	^{13}C-NMR (125 MHz)
1		159.3
2	6.07 (t, 2.2)	100.9
3		159.3
4	6.12 (d, 2.2)	107.9
5		146.3
6	6.12 (d, 2.2)	107.9
1’	2.43 (t, 7.6)	37.0
2’	1.56 (m)	32.5
3’	1.26-1.34	30.6
4’	1.24-1.28	30.6
5’	1.24-1.28	30.6
6’	1.24-1.28	30.6
7’	1.24-1.28	30.6
8’	1.24-1.28	30.6
9’	1.24-1.28	30.6
10’	1.24-1.28	30.6
11’	1.24-1.28	30.6
12’	1.24-1.28	30.6
13’	1.24-1.28	33.1
14’	1.24-1.28	23.7
15’	0.90 (t, 6.8)	14.4

Table S2. NMR resonance assignments for compound **2** in CD_3OD.

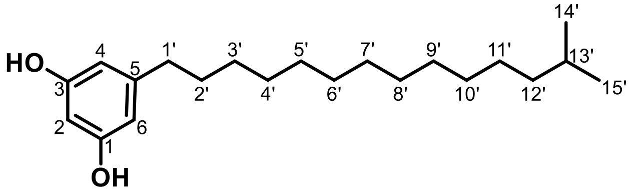

Number	^{1}H-NMR (500 MHz)	^{13}C-NMR (125 MHz)
1		159.3
2	6.08 (t, 2.2)	100.9
3		159.3
4	6.12 (d, 2.2)	107.9
5		146.3
6	6.12 (d, 2.2)	107.9
1’	2.43 (t, 7.6)	37.0
2’	1.56 (m)	32.4
3’	1.26-1.34	30.7
4’	1.24-1.28	30.7
5’	1.24-1.28	30.7
6’	1.24-1.28	30.7
7’	1.24-1.28	30.7
8’	1.24-1.28	30.7
9’	1.24-1.28	30.7
10’	1.24-1.28	30.7
11’	1.24-1.28	28.5
12’	1.18 (m)	40.2
13’	1.52 (m)	29.2
14’	0.88 (t, 6.6)	23.0
15’	0.88 (t, 6.6)	23.0

Table S3. NMR resonance assignments for compound **6** in D_2O.

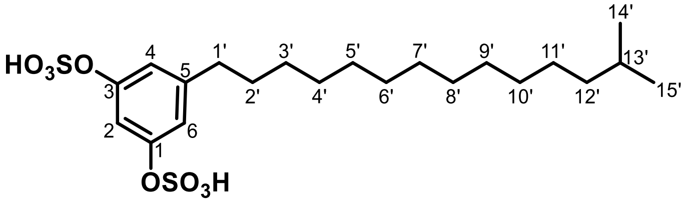

Number	^{1}H-NMR (500 MHz)	^{13}C-NMR (125 MHz)
1		151.6
2	7.20, s	112.1
3		151.6
4	7.03, s	119.2
5		146.2
6	7.03, s	119.2
1'	2.62 (t, 7.6)	35.4
2'	1.58 (m)	31.1
3'	1.24-1.28	29.4-31.1
4'	1.24-1.28	29.4-31.1
5'	1.24-1.28	29.4-31.1
6'	1.24-1.28	29.4-31.1
7'	1.24-1.28	29.4-31.1
8'	1.24-1.28	29.4-31.1
9'	1.24-1.28	29.4-31.1
10'	1.24-1.28	29.4-31.1
11'	1.24-1.28	27.9
12'	1.17 (m)	39.1
13'	1.51(m)	32.0
14'	0.87(d, 7.1)	22.5
15'	0.87(d, 7.1)	22.5

Table S4. NMR resonance assignments for compound **7** in CD_3OD.

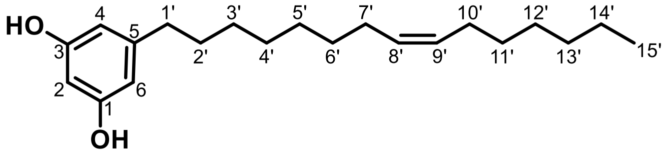

Number	^{1}H-NMR (500 MHz)	^{13}C-NMR (125 MHz)
1		159.3
2	6.08 (t, 2.2)	100.9
3		159.3
4	6.12 (d, 2.2)	107.9
5		146.3
6	6.12 (d, 2.2)	107.9
1’	2.43 (t, 7.6)	37.0
2’	1.56 (m)	32.4
3’	1.26-1.36	30.4
4’	1.26-1.36	30.4
5’	1.26-1.36	30.4
6’	1.26-1.36	30.4
7’	2.03 (m)	28.1
8’	5.34 (m)	130.8
9’	5.34 (m)	130.8
10’	2.03 (m)	28.1
11’	1.26-1.36	30.4
12’	1.26-1.36	30.4
13’	1.26-1.36	32.9
14’	1.26-1.36	23.7
15’	0.90 (t, 6.8)	14.4

Table S5. Primers used in this study.

Primer	Sequence (5' → 3')
pACYC_*adpPKS*-F	AGCCATCACCATCATCACCACAGCCAGGATatgcatctcgaagagaaaatcg
pACYC_*adpPKS*-R	ATACGATTACTTTCTGTTCGACTTAAGCAtcaggaccggcgcagcaggg
pHis8_*adpST*-F	gtctggttccgcgtggttcccatggcggatccatgcccaccccgacctc
pHis8_*adpST*-R	tggtggtggtgctcgagtgcggccgcaagctttcatgcgctgctccttg
pMAL_*adpST*-F	ATCGAGGGAAGGATTTCACAtatgcccaccccgacctc
pMAL_*adpST*-R	ATTACCTGCAGGGAATTCGGAggaaccacgcggaaccagtcatgcgctgctccttg
pCAP01_*adpPKS-F*	atggtttctacaaagatcgactagtaatgcatctcgaagagaaaatcg
pCAP01_*adpPKS-R*	tacaggtacctcaagtctcgaggtcaggaccggcgcagcaggg
pKY01_*adpST*-F	agcggtccaacgcaccccaggaggtccccatatgatgcccaccccgacctc
pKY01_*adpST*-R	gcgcgcggccgcggatcctctagtaagctttcatgcgctgctccttg
pCAP01_*adp*_full-F	tggtttctacaaagatcgactagtagcgagcaccgaataacacca
pCAP01_*adp*_full-R	tacaggtacctcaagtctcgaatgctcaggcagccgcat
